# TGF-β1 mediates hypoxia-preconditioned olfactory mucosa mesenchymal stem cells improved neural functional recovery in Parkinson’s disease models and patients

**DOI:** 10.1186/s40779-024-00550-7

**Published:** 2024-07-22

**Authors:** Yi Zhuo, Wen-Shui Li, Wen Lu, Xuan Li, Li-Te Ge, Yan Huang, Qing-Tao Gao, Yu-Jia Deng, Xin-Chen Jiang, Zi-Wei Lan, Que Deng, Yong-Heng Chen, Yi Xiao, Shuo Lu, Feng Jiang, Zuo Liu, Li Hu, Yu Liu, Yu Ding, Zheng-Wen He, De-An Tan, Da Duan, Ming Lu

**Affiliations:** 1 Hunan Provincial Key Laboratory of Neurorestoratology, 921 Hospital of Joint Logistics Support Force People’s Liberation Army of China, (the Second Affiliated Hospital of Hunan Normal University), Changsha, 410003 China; 2 Department of Neurosurgery, the Affiliated Cancer Hospital of Xiangya School of Medicine, Central South University/Hunan Cancer Hospital, Changsha, 410000 China; 3 The National & Local Joint Engineering Laboratory of Animal Peptide Drug Development, College of Life Sciences, Hunan Normal University, Changsha, 410006 China; 4 Department of Neurology, the Second Xiangya Hospital, Central South University, Changsha, 410013 China; 5 NHC Key Laboratory of Birth Defect for Research and Prevention, Hunan Provincial Maternal and Child Health Care Hospital, Changsha, 410008 China; 6 First Clinical Department of Changsha Medical University, Changsha, 410219 China

**Keywords:** Parkinson’s disease (PD), Hypoxia-preconditioned, Olfactory mucosa mesenchymal stem cells (OM-MSCs), Transforming growth factor-β1 (TGF-β1), Microglia, PI3K/Akt signaling pathway, Immune regulation, Autophagy

## Abstract

**Background:**

Parkinson’s disease (PD) is a neurodegenerative disorder characterized by the degeneration of dopaminergic neurons in the substantia nigra (SN). Activation of the neuroinflammatory response has a pivotal role in PD. Mesenchymal stem cells (MSCs) have emerged as a promising therapeutic approach for various nerve injuries, but there are limited reports on their use in PD and the underlying mechanisms remain unclear.

**Methods:**

We investigated the effects of clinical-grade hypoxia-preconditioned olfactory mucosa (hOM)-MSCs on neural functional recovery in both PD models and patients, as well as the preventive effects on mouse models of PD. To assess improvement in neuroinflammatory response and neural functional recovery induced by hOM-MSCs exposure, we employed single-cell RNA sequencing (scRNA-seq), assay for transposase accessible chromatin with high-throughput sequencing (ATAC-seq) combined with full-length transcriptome isoform-sequencing (ISO-seq), and functional assay. Furthermore, we present the findings from an initial cohort of patients enrolled in a phase I first-in-human clinical trial evaluating the safety and efficacy of intraspinal transplantation of hOM-MSC transplantation into severe PD patients.

**Results:**

A functional assay identified that transforming growth factor-β1 (TGF-β1), secreted from hOM-MSCs, played a critical role in modulating mitochondrial function recovery in dopaminergic neurons. This effect was achieved through improving microglia immune regulation and autophagy homeostasis in the SN, which are closely associated with neuroinflammatory responses. Mechanistically, exposure to hOM-MSCs led to an improvement in neuroinflammation and neural function recovery partially mediated by TGF-β1 via activation of the anaplastic lymphoma kinase/phosphatidylinositol-3-kinase/protein kinase B (ALK/PI3K/Akt) signaling pathway in microglia located in the SN of PD patients. Furthermore, intraspinal transplantation of hOM-MSCs improved the recovery of neurologic function and regulated the neuroinflammatory response without any adverse reactions observed in patients with PD.

**Conclusions:**

These findings provide compelling evidence for the involvement of TGF-β1 in mediating the beneficial effects of hOM-MSCs on neural functional recovery in PD. Treatment and prevention of hOM-MSCs could be a promising and effective neuroprotective strategy for PD. Additionally, TGF-β1 may be used alone or combined with hOM-MSCs therapy for treating PD.

**Supplementary Information:**

The online version contains supplementary material available at 10.1186/s40779-024-00550-7.

## Background

Parkinson’s disease (PD) is a neurodegenerative disease that is characterized by the degeneration of dopaminergic neurons in the substantia nigra (SN) [1, 2]. The primary clinical manifestations of PD include quiescent tremors, myotonia, tardiness, and abnormal postural gait [3, 4]. Immune system dysregulation has also emerged as a crucial factor in PD susceptibility and progression, garnering increasing attention over the past decade. PD is marked by the death of dopaminergic neurons containing Lewy bodies (LBs), which mainly consist of α-synuclein (α-Syn) aggregates in the SN and serve as definitive diagnostic features during postmortem examinations [5, 6]. Throughout disease progression, misfolded α-Syn becomes the major constituent of LBs and spreads to various brain regions in a prion-like manner [7]. Microglia represents the first line of defense in the brain’s immune system and plays a pivotal role in the central nervous system (CNS)’s inflammatory response [8, 9]. Misfolded α-Syn protein binds to microglial cell surface receptors activating them and triggering persistent inflammatory responses [10–12]. Due to a limited understanding of PD etiology, symptomatic clinical management traditionally relies on pharmacologic interventions, such as levodopa and neurosurgical procedures. However, long-term oral administration of levodopa can lead to adverse effects while drug resistance usually renders this treatment ineffective. Although these approaches provide partial relief from symptoms for patients with PD, neuronuclear destruction caused by deep brain stimulation surgeries may result in irreversible neurological damage [13, 14].

With the advancement of stem cell technology, nerve repair and regeneration have become feasible. Stem cell transplantation has also shown promising results in the treatment of PD. Mesenchymal stem cells (MSCs), which are adult stem cells with paracrine, immune regulatory, and multidirectional differentiation potential [15–17], were previously hypothesized to primarily address the degeneration of dopaminergic neurons underlying PD. Most researchers believe that MSCs’ therapeutic effect on PD is attributed to their ability to replace damaged cells [18, 19]. However, research involving MSCs has shifted its focus from cell replacement to multitargeted therapy such as paracrine signaling and immune regulation [20, 21]. Exploring immunomodulation in PD presents an opportunity for identifying novel therapeutic targets and strategies for mitigating or reversing neurodegenerative changes. In 2010, researchers discovered that olfactory mucosal (OM)-derived MSCs isolated from human nasal mucosa represent a superior source of MSCs [22]. Since 2015, our research team has been conducting a comprehensive investigation involving autologous human OM-MSCs and successfully developed a complete culture system [23].

The success of MSC therapy depends on the survival rate of cells, the number of homing cells, and the immunomodulatory effects after implantation in vivo. The microenvironment at the site of injury for inflammatory immune diseases is characterized by excessive inflammation, oxidative stress, and hypoxia, which pose significant challenges to the efficacy of MSCs [23]. During in vitro amplification, the MSCs were cultured under normoxic conditions with a serum concentration ranging from 10 to 20%. However, upon implantation into disease models or patients, MSCs encounter hypoxic or ischemic microenvironments. Developing an optimized culture protocol for hypoxia-preconditioned MSCs that enhances their survival rate, homing capacity, and paracrine effect represents a promising avenue for research aimed at overcoming some of the challenges associated with MSC-based therapy.

We conducted a groundbreaking study to investigate the effects of clinical-grade hypoxia-preconditioned OM-MSCs (hOM-MSCs) on the recovery of neural functional in PD models and patients, as well as their preventive effects on PD mouse models. To assess improvement in neuroinflammatory response and neural functional recovery induced by hOM-MSCs exposure, we employed single-cell RNA sequencing (scRNA-seq), assay for transposase accessible chromatin with high-throughput sequencing (ATAC-seq) combined with full-length transcriptome isoform-sequencing (ISO-seq), and functional assay. Furthermore, we present the findings from an initial cohort of patients enrolled in a phase I first-in-human clinical trial evaluating the safety and efficacy of intraspinal transplantation of hOM-MSC transplantation into severe PD patients.

## Materials and methods

### Ethics statement

The experiments were conducted in accordance with the approved guidelines and received approval from both the institutional ethical committee and the animal care committee at Hunan Normal University (2020–390). Human nasal mucosa biopsies were obtained with informed consent, and all experiments were granted approval by the Ethics Committee at the Second Affiliated Hospital of Hunan Normal University (2020–390). Furthermore, all clinical investigations have been conducted according to the principles outlined in the Declaration of Helsinki.

### Acquisition, culture and preparation of clinical-grade hOM-MSCs

OM-MSCs were obtained from PD patients who volunteered at the Second Affiliated Hospital of Hunan Normal University. Three days prior to collecting the OM tissue, chloramphenicol was administered in the patient’s nasal cavity for a duration of 3 d. The collection tube for OM was prepared and inspected for any signs of damage. Before obtaining the tissue, gauze sheets were soaked in 1.2 – 1.5% tetracaine and saline solution, then inserted into the patient’s nasal cavity twice for surface local anesthesia with each session lasting 5 – 8 min. The ethmoid forceps were used to clip the mucosal tissue on the lateral side of the upper part of the middle turbinate, resulting in a tissue size of approximately 3 mm^3^. Subsequently, this tissue block was carefully placed into a collection tube using a syringe needle with relevant information on said tube, immediately after which it was transferred into an OM constant temperature transportation box. Finally, the collection tube was removed from refrigeration at 4 ℃ and temporarily stored.

The OM samples were triple rinsed in DMEM/F12 (v/v = 1:1) mixed medium (Gibco, Grand Island, NY, USA) supplemented with 200 U/ml penicillin and 200 U/ml streptomycin to remove any residual blood. Subsequently, the samples were transferred into DMEM/F12 medium containing 10% autoserum, along with 100 U/ml penicillin and 100 U/ml streptomycin. The minced tissue was obtained by using ophthalmic scissors and cut into pieces of approximately 0.5 mm^3^ size. These tissue fragments were then centrifuged to remove the supernatant. The resulting pellets were seeded in corning culture flasks (Corning, NY, USA). Once the cells reached full confluence within the flask surface area, they were trypsinized and passaged. The hOM-MSCs were cultured under a 3% oxygen concentration for a duration of 48 h.

To ensure the quality, safety, and efficacy of hOM-MSCs preparations, it is imperative to further standardize the procedures for quality inspection of hOM-MSCs. The detection procedure primarily involves characterizing hOM-MSCs utilizing flow cytometry and immunofluorescence techniques to quantify the expression of cell surface markers (CD34, CD45, CD44, CD73, CD90, CD105, CD133, CD146) as well as specific markers [stromal cell antigen 1 (STRO-1) and Nestin]. The tests for bacteria, fungi, mycoplasma, and exogenous viruses yielded negative results, while the concentration of endotoxin was found to be below the prescribed threshold. Biological activity detection mainly focuses on assessing cell viability, proliferation capacity, and differentiation potential. For fresh cells, a minimum acceptable viability rate is set at 90%, while for liquid nitrogen frozen cells, it is 80%. Cells were inoculated at a density of 1 × 10^6^ cells/T75 vials and harvested after 48 h of culture for digestion and counting. The obtained cell count should range from 1.5 to 2.5 times of the initial inoculation count. These cells possess the capability to differentiate into adipocytes, osteoblasts, chondrocytes, and neurons. Additionally, 10 ml of 0.9% normal saline should be added evenly to each tube containing the centrifuged hOM-MSCs and distributed. Centrifuge for 5 min, perform three rounds of cleaning according to the specified requirements, enumerate the cells, discard the supernatant, add 2 ml of 0.9% normal saline, and distribute uniformly to obtain the final cell preparation. Transfer it into a specialized transport incubator at 4 ℃ for experimental research and clinical trials.

### Animals

Eighty-four male C57BL/6 mice, aged 10 – 12 weeks, were procured from Hunan SJA Laboratory Animal Co., Ltd. (Hunan, China) and housed in a facility with controlled temperature, humidity, and a 12 h light/dark cycle. Food and water were provided ad libitum. All procedures were conducted in accordance with the Guide for the Care and Use of Laboratory Animals after receiving approval from the Ethics Committee at the Second Affiliated Hospital of Hunan Normal University (Ethical Approval Document 2020–390).

### PD cell model and intervention

BV2 cells (1 × 10^5^; SCSP-5014, National Collection of Authenticated Cell Cultures, China) were planted in the bottom layer of the upper chamber of the Transwell, while SH-SY5Y cells (1 × 10^5^; GDC0311, China Center for Type Culture Collection, China) were planted in the lower chamber. According to the experimental requirements, BV2 cells and SH-SY5Y cells were positioned differently. After 24 h, activated α-Syn (250 nmol/L; ab218819, Abcam, USA) was added to the culture medium and incubated for 1 h in a carbon dioxide incubator [24]. To prevent internalization of α-Syn into MSCs, the lower chamber of the Transwell was rinsed three times with fresh medium. Subsequently, 1 × 10^5^ OM-MSCs cultured under normoxic or hypoxic conditions were co-cultured with BV2 and SH-SY5Y cells for 12 h.

In this study, OM-MSCs were evaluated for their neuroprotective effects in a PD model, resulting in four groups: control, model group (α-Syn), normoxic OM-MSCs intervention group (normoxia), and hypoxic OM-MSCs intervention group (hypoxia). The SH-SY5Y cells in each group were fluorescent-stained with neuron-specific nuclear protein (NeuN), a marker of neurons, and BAX, a marker of apoptosis. The neuroprotective effect of hOM-MSCs mediated by TGF-β1 on neurons in PD cell models was investigated, along with the ability of microglia to regulate immune responses and clear α-Syn. The experiment was divided into four intervention groups: hOM-MSCs, hOM-MSCs + shRNA TGF-β1, TGF-β1, and TGF-β1 + siRNA ALK.

The neuroprotective effect of hOM-MSCs mediated by TGF-β1 on neurons in PD cell models was investigated, along with the ability of microglia to regulate immune responses and clear α-Syn. The experiment was divided into four intervention groups: hOM-MSCs, hOM-MSCs + shRNA TGF-β1, TGF-β1, and TGF-β1 + siRNA ALK. Immunologic regulation by hOM-MSCs via activation of the PI3K-Akt signaling pathway in microglia cell secretion of TGF-β1 was determined. The experiment was divided into four groups: control, α-Syn, hOM-MSC, and hOM-MSC + shRNA TGF-β1. The role of human recombinant protein TGF-β1 in immune regulation was investigated in microglial cells through the activation of the PI3K-Akt signaling pathway, and the experiment was divided into four groups: Control, α-Syn, TGF-β1, and TGF-β1 + siRNA ALK.

### PD mouse model and cell transplantation

Neurotoxicity was induced by 1-methyl-4-phenyl-1,2,3,6-tetrahydropyridine (MPTP; M0896, Sigma, USA) combined with probenecid, as previously reported [25]. Briefly, male C57BL/6 mice were intraperitoneally injected with 25 mg/kg MPTP and 250 mg/kg probenecid (P8761, Sigma, USA) once every 3.5 d for a total of 10 injections. The sham operation group received an equivalent volume of normal saline via intraperitoneal injection. Then, behavioral assessments were conducted in order to determine the success of the PD model. One week following the final MPTP injection, a stereotactic injection of 1 × 10^5^ cells/10 μl phosphate buffer saline (PBS) was administered into the right lateral ventricle at coordinates -0.6 mm posterior, -1.5 mm lateral, and -1.7 mm ventral to the bregma. The sham operation group was injected with an equal amount of normal saline at the corresponding site.

The prophylactic and therapeutic effects of hOM-MSCs on mouse models of PD were evaluated, followed by the establishment of subsequent cohorts: sham operation (sham), model (PD + PBS), normoxic OM-MSCs treatment (PD + nOM-MSCs), hOM-MSCs treatment (PD + hOM-MSCs), and hOM-MSCs prevention (Pre + hOM-MSCs). The therapeutic potential of TGF-β1 in a mouse model of PD was investigated by examining its binding to the ALK receptor on microglial cell membranes, followed by modulation of hOM-MSCs. The experiment consisted of 4 treatment groups PD + hOM-MSCs, PD + hOM-MSCs + shRNA TGF-β1, PD + TGF-β1, and PD + AAV ALK + TGF-β1. The noteworthy aspect is that each group in the aforementioned experiment consisted of 12 mice.

The immunologic regulation of hOM-MSCs through activation of the PI3K-Akt signaling pathway was further investigated in microglia cells that secret TGF-β1. The experiment was divided into 4 groups: sham, PD + PBS, PD + hOM-MSCs, and PD + hOM-MSCs + shRNA TGF-β1. The phosphorylation status of the PI3K-Akt signaling pathway, the mTOR-mediated regulation of cellular activity and autophagic homeostasis, as well as the levels of p50 and p65 involved in immune-inflammatory responses, were assessed in the experimental groups. Furthermore, the experiment was divided into four groups to explore the role of human recombinant protein TGF-β1 in immune regulation by activating the PI3K-Akt signaling pathway in microglial cells: sham, PD + PBS, PD + TGF-β1, and PD + AAV ALK + TGF-β1. The phosphorylation status of the PI3K-Akt and mTOR signaling pathway, as well as the levels of p50 and p65 protein expression, were evaluated in the experimental groups. The noteworthy aspect is that each group in the aforementioned experiment consisted of 12 mice.

### Clinical trial method steps and evaluation

This is a clinical trial investigating the transplantation of hOM-MSCs for PD. The sample consisted of 5 patients, all of whom were volunteers recruited for clinical trials and diagnosed with PD, aged between 50 and 80 years old., with a disease duration exceeding 5 years, the Hoehn and Yahr grade of 3 or above, and inadequate response to drug treatment, thus meeting the eligibility criteria. No control group was included in this study. The participants received an intrathecal injection of autologous serum-cultured hOM-MSCs derived from their own OM. The study protocol was approved by the Ethics Committee at the Second Affiliated Hospital of Hunan Normal University (2020–390) and conformed to the Declaration of Helsinki guidelines. All participants provided written informed consent before recruitment. This study has been registered on the Chinese Clinical Trial Registry (Registration No: ChiCTR2100055021, https://www.chictr.org.cn/).

Prospective data have been collected, including the UPDRS total score, Hoehn and Yahr rating scale, and Schwab and England daily activity scale. CSF and serum samples were tested for DA, TGF-β1, CD206, IL-1β, TNF-α, IL-4, and IL-10. The CSF and serum samples were collected from 4 patients before and after treatment, followed by a series of relevant assays to measure inflammatory cytokines. The protein expression levels of immunoregulatory factor TGF-β1 and dynamic markers of microglia inflammatory phenotype IL-1β and CD206 in the CSF were assessed by Western blotting analysis. The concentrations of DA neurotransmitters and related inflammatory factors in both CSF and serum samples from patients were analyzed using ELISA. A video recording was obtained for a comparative assessment of treatment efficacy.

#### Inclusion criteria

The following inclusion criteria were applied to qualify patients for enrollment: 1) age range between 50 and 80 years; 2) diagnosis of less than 20 years; 3) patients exhibiting inadequate response to oral medication; 4) the patients themselves or their legal guardian must provide informed consent and sign the voluntary form in order to participate in this clinical trial.

#### Exclusion criteria

Patients were excluded from this study if they: 1) presented with CNS inflammatory diseases such as viral or parasitic meningitis, chronic decompensated psychosis, dementia, CNS tumors, coagulation disorders, thrombocytopenia, active infectious diseases including human immunodeficiency virus, syphilis, hepatitis B or hepatitis C infections; 2) had a previous medical history of tumor diseases; exhibited a highly allergic constitution or history of severe allergies; or 3) suffered from mental or psychological conditions that hindered treatment cooperation.

#### Exit criteria

If the patient meets the termination criteria specified in the protocol during the course of the trial, such as vital organ dysfunction, drug allergic reaction, poor compliance, aggravation of the condition, or serious adverse reactions, it is necessary to stop the experimental cell therapy or consider alternative treatment methods. If the patient experiences poor therapeutic effects, cannot tolerate adverse reactions, wishes to pursue alternative treatment methods, or withdraws from the trial without any reason. The researcher may allow the participant to withdraw from further participation in the experiment.

#### Steps for transplanting cells

The hOM-MSCs preparation (as described above) involves transplanting cells 5 × 10^7^/time, 2 – 3 times over a treatment course of 14 – 21 d. Patients receive intraspinal injections of the transplanted cells, following specific steps: the patient lies on his side on a hard plank bed, with his back perpendicular to the bed surface. The puncture point is the space between the spinous processes of the 3rd to 4th lumbar vertebrae. Wear sterile gloves, routinely disinfect the skin, cover the hole with a towel, and use 2% lidocaine for local anesthesia from the skin to the intervertebral ligament. The surgeon fixes the skin at the puncture point with his left hand, holds the puncture needle in his right hand, and inserts the needle slowly in a direction perpendicular to the back, with a depth of about 4 – 6 cm. Collect cerebrospinal fluid (CSF) for testing in accordance with the specified requirements. Aspirate the prepared cell suspension using a syringe and gradually administer it into the spinal canal. Following injection, insert the needle core, remove the puncture needle, cover with sterile gauze, and secure with adhesive tape.

#### Safety assessment

General item evaluation includes routine blood, urine, and stool routine, liver and kidney function electrolytes, blood glucose levels, blood lipid profile, as well as a complete coagulation panel. Assessment of potential side effects encompasses exacerbation of symptoms, fever, rash, headache, dizziness, nausea and vomiting, diarrhea, muscle pain, etc.

#### Efficacy evaluation

Neurological assessment includes the total unified PD rating scale (UPDRS) score, Hoehn and Yahr rating scale, and Schwab and England daily activity scale. Additionally, CSF and serum samples will be tested for dopamine (DA), transforming growth factor-β1 (TGF-β1), CD206, interleukin (IL)-1β, tumor necrosis factor (TNF)-α, IL-4, and IL-10. A video recording will be taken to compare treatment efficacy.

In addition to the aforementioned materials and methods, a comprehensive description of supplementary materials and methods can be found in Additional file 1: Materials and methods.

### Statistical analysis

GraphPad Prism 8.0 software was used to analyze the experimental data, and the experimental data were expressed as mean ± standard error of the mean (mean ± SEM). ANOVA and paired *t*-tests were used to analyze the sample data. *P* < 0.05 indicates statistical significance.

## Results

### Identification and biological characteristics of OM-MSCs

Our culture system for OM-MSCs successfully produces spindle-shaped cells with a radial arrangement under light microscopy (Additional file 1: Fig. S1a). The intracellular location of STRO-1 (a marker for stromal cells) and Nestin (a marker for stem cells) was detected using immunofluorescence (Additional file 1: Fig. S1b, c). Upon staining the cells induced by lipogenic inducers with Oil Red O, red lipid droplets were observed in the cytoplasm, suggesting that adipogenic differentiation had been achieved (Additional file 1: Fig. S1d). After induction by an osteogenic inducer, Alizarin Red staining demonstrated the formation of red mineralized nodules in the cells, indicating successful osteogenic differentiation (Additional file 1: Fig. S1e). The above steps are critical for characterizing OM-MSCs since they provide evidence of their potential for dividing into adipocytes and bone cells and confirm their ability to repair and regenerate. OM-MSCs showed numerous microvilli on their surface under a scanning electron microscope (SEM) (Additional file 1: Fig. S1f). Observations of cells by transmission electron microscopy (TEM) revealed that OM-MSCs were in a relatively active phase (Additional file 1: Fig. S1g) characterized by two nuclei present in each cell; both nuclei exhibited round or oval with prominent nucleoli. In addition, the rough endoplasmic reticulum and mitochondria were observed among other organelles. Finally, flow cytometry assays showed high expression levels of CD44, CD73, CD90, CD105, CD133, and CD146 in OM-MSCs, while OM-MSCs did not express CD34 and CD45, with the purity rate of OM-MSCs exceeding 97% (Additional file 1: Fig. S1h).

### hOM-MSCs enhanced mitochondrial function in neurons and the immunomodulation of microglia in PD cell model

**Fig. 1:**
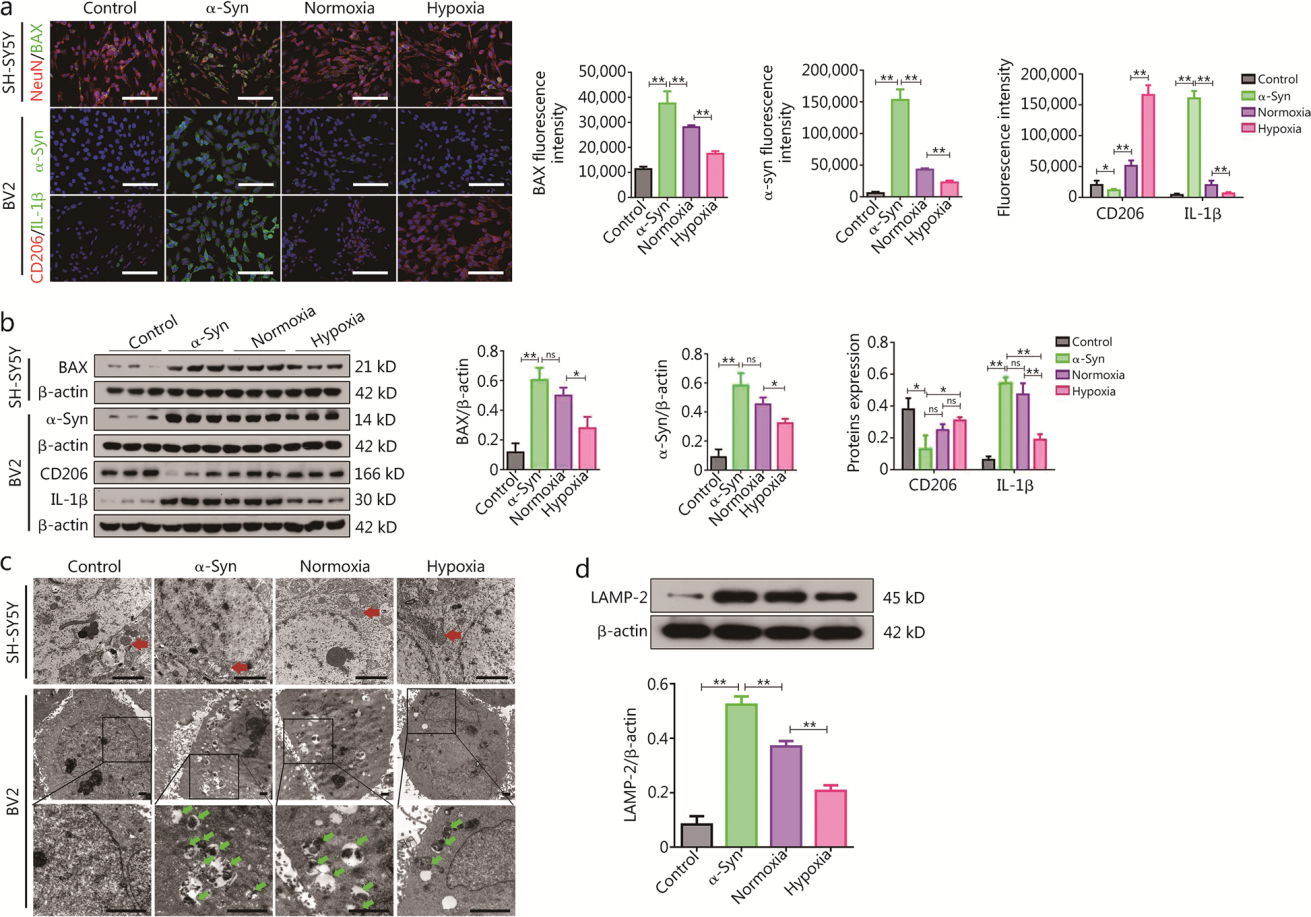
The hOM-MSCs enhanced mitochondrial function in neurons and the immunomodulation of microglia in the PD cell model. **a** Exemplary immunofluorescence micrograph showing nuclei (DAPI, blue), NeuN, and BAX expression in SH-SY5Y cells, and nuclei (DAPI, blue), α-Syn, CD206, and IL-1β expression in BV2 cells (scale bars = 40 μm). **b** Western blotting measuring BAX protein expression in SH-SY5Y cells, and α-Syn (α-synuclein), IL-1β, and CD206 protein expression in BV2 cells. **c** TEM showing the mitochondria morphology and ultrastructure (red arrow) in SH-SY5Y cells, and the formation of autophagosomes (green arrow) in BV2 cells (scale bars = 2 μm). **d** Western blotting measuring LAMP-2 protein expression in BV2 cells. Data are represented as mean ± SEM. ^*^P < 0.05, ^**^P < 0.01, ns non-significant. NeuN neuron-specific nuclear protein, DAPI 4’,6-diamidino-2-phenylindole, IL-1β interleukin-1β, LAMP-2 lysosome-associated membrane protein-2, hOM-MSCs hypoxia-olfactory mucosa mesenchymal stem cells, PD Parkinson’s disease, CD recombinant cluster of differentiation

The immunofluorescence results revealed that compared to the control group, the α-Syn group exhibited a remarkable increase in SH-SY5Y cell apoptosis, accompanied by an alteration in cytoskeletal morphology and a reduction in axon length. In the normoxia group, apoptosis was significantly decreased and cytoskeleton morphology was improved. In addition, the hypoxia group showed the most significant decrease in apoptosis, improvement in cytoskeleton morphology, and elongation of the axon (Fig. 1a; Additional file 1: Fig. S2a). These findings were further supported by Western blotting and flow cytometry analysis (Fig. 1b; Additional file 1: Fig. S2b), which confirmed that OM-MSCs have a protective effect on neurons, and this neuroprotective function is significantly enhanced in hOM-MSCs.

OM-MSCs exert a protective role on neuronal mitochondria in a PD model by assessing membrane potential [5,5',6,6'-tetrachloro-1,1',3,3'-tetraethylbenzimidazolcarbocyanine iodide (JC-1)] and reactive oxygen species (ROS), which are indicators of mitochondrial function. In the α-Syn group, there was a significant decrease in JC-1 fluorescence intensity, indicating a reduced mitochondrial membrane potential, and an increase in ROS production compared to the control group. On the other hand, under normoxic conditions, JC-1 fluorescence intensity increased and ROS production decreased. However, hypoxia-induced the most significant increase in JC-1 fluorescence intensity and decrease in ROS production (Additional file 1: Fig. S2c). Additionally, TEM was used to examine the changes in mitochondrial ultrastructure. The results showed that the mitochondria in the α-Syn group exhibited significant swelling and disorganization compared to the control group. In contrast, the mitochondria appeared to improve under normoxic conditions, but their recovery was most remarkable following hypoxia treatment (Fig. 1c). In conclusion, OM-MSCs protect neuronal mitochondrial function, and their protective effect is enhanced by hypoxia pretreatment.

The aim of this study was to investigate the efficacy of OM-MSCs in eliminating α-Syn from activated microglia cells. Immunofluorescence findings revealed that α-Syn expression in BV2 cells was upregulated in the α-Syn group compared to the control group. As compared to the normoxia group, its expression decreased in the hypoxia group (Fig. 1a; Additional file 1: Fig. S2d). Western blotting analyses (Fig. 1b) further supported this finding which indicated OM-MSCs possess scavenging abilities on microglia cells, which are significantly enhanced under hypoxic conditions. The immunomodulatory effect of OM-MSCs on activated microglia cells, specifically their transition from the proinflammatory M1 phenotype to the anti-inflammatory M2 phenotype, was investigated by assessing the expression levels of the anti-inflammatory marker CD206 and proinflammatory marker IL-1β in BV2 cells. The expression of CD206 was significantly downregulated, while the expression of IL-1β was significantly upregulated in BV2 cells of the α-Syn group compared to the control group upon Western blotting. Conversely, in the normoxia group, CD206 expression was increased and IL-1β expression was decreased, with even greater effects observed in the hypoxia group (Fig. 1a; Additional file 1: Fig. S2e). These findings were also confirmed by Western blotting analysis (Fig. 1b), indicating that OM-MSCs exert an immunomodulatory effect by promoting the transformation of microglia from M1 to M2 phenotype, with hOM-MSCs showing a more pronounced immunomodulatory effect.

In a PD model, the regulatory effect of OM-MSCs on microglial autophagy homeostasis was further investigated in a PD model. The formation of autophagosomes was observed by TEM in BV2 cells, and the expression of lysosome-associated membrane protein-2 (LAMP-2) was detected by Western blotting. The TEM results revealed a conspicuous absence of autolysosome formation in the control group, whereas an astonishing surge in autolysosomes was observed in the model group; however, the structure of lysosomes within the autophagosome was severely damaged and exhibited incomplete membranes. In the normoxia group, there were formed but decreased numbers of autolysosomes, and an improvement in lysosomal structure compared to the model group was observed. In contrast, a significant reduction in lysosome formation occurred in the hypoxia group; nevertheless, there were still present autopolysomes with intact lysosomal structures (Fig. 1c). By analyzing Western blots, we confirmed the validity of this finding (Fig. 1d), which showed that hOM-MSCs ameliorated excessive autophagic response, and maintained autophagy homeostasis to facilitate degradation and clearance of α-Syn in microglia.

### hOM-MSCs facilitated the recovery of nerve function and the immunomodulation of microglia in PD mouse model

The chronic PD mouse model was established through intraperitoneal injection of MPTP in combination with probenecid. The experiment included sham and PD model groups. Neurologic function scores indicated that the PD group showed significant reductions in the total distance, average speed, and central time, as measured by the open field test, while results from the Tatarod test passive motor function scores exhibited significant reductions in both the total distance and latent period (Additional file 1: Fig. S3a, b). Tyrosine hydroxylase (TH) is a rate-limiting enzyme in DA synthesis, whose expression is proportional to the number of dopaminergic neurons. Immunohistochemistry was used to detect changes in TH^+^ cells within the SN of mice; TH^+^ staining was significantly reduced in the PD group compared to the sham group (Additional file 1: Fig. S3c). The DA reuptake inhibitor, 2β-carbomethoxy-3β-(4-fluorophenyl)-(N-11C-methyl) tropane (CFT), which is based on the phenyltolane structure, and 11C-CFT have a high affinity with presynaptic membrane dopamine transporter (DAT), reflecting the function of dopaminergic neurons in the substantia striatum pathway. CFT-PET brain imaging was used to observe the distribution of DAT in different regions of the brain, revealing a significantly reduced distribution of DAT in the bilateral SN striatum and hippocampus regions of the PD group, indicating impaired dopaminergic neuron function (Additional file 1: Fig. S3d). The proposition of a neuroimmune microenvironment is put forward to explain the onset and progression of PD. Western blotting analysis was performed to detect proteins in the SN, including α-Syn, proinflammatory cytokines [major histocompatibility complex class II (MHC-II) and IL-1β], anti-inflammatory cytokines (CD206), and T cell phenotypes (CD8^+^ and CD4^+^). The findings revealed significant upregulation of α-Syn, MHC-II, IL-1β, CD8^+^, and CD4^+^ in the PD group, while CD206 exhibited remarkable downregulation (Additional file 1: Fig. S3e).

**Fig. 2:**
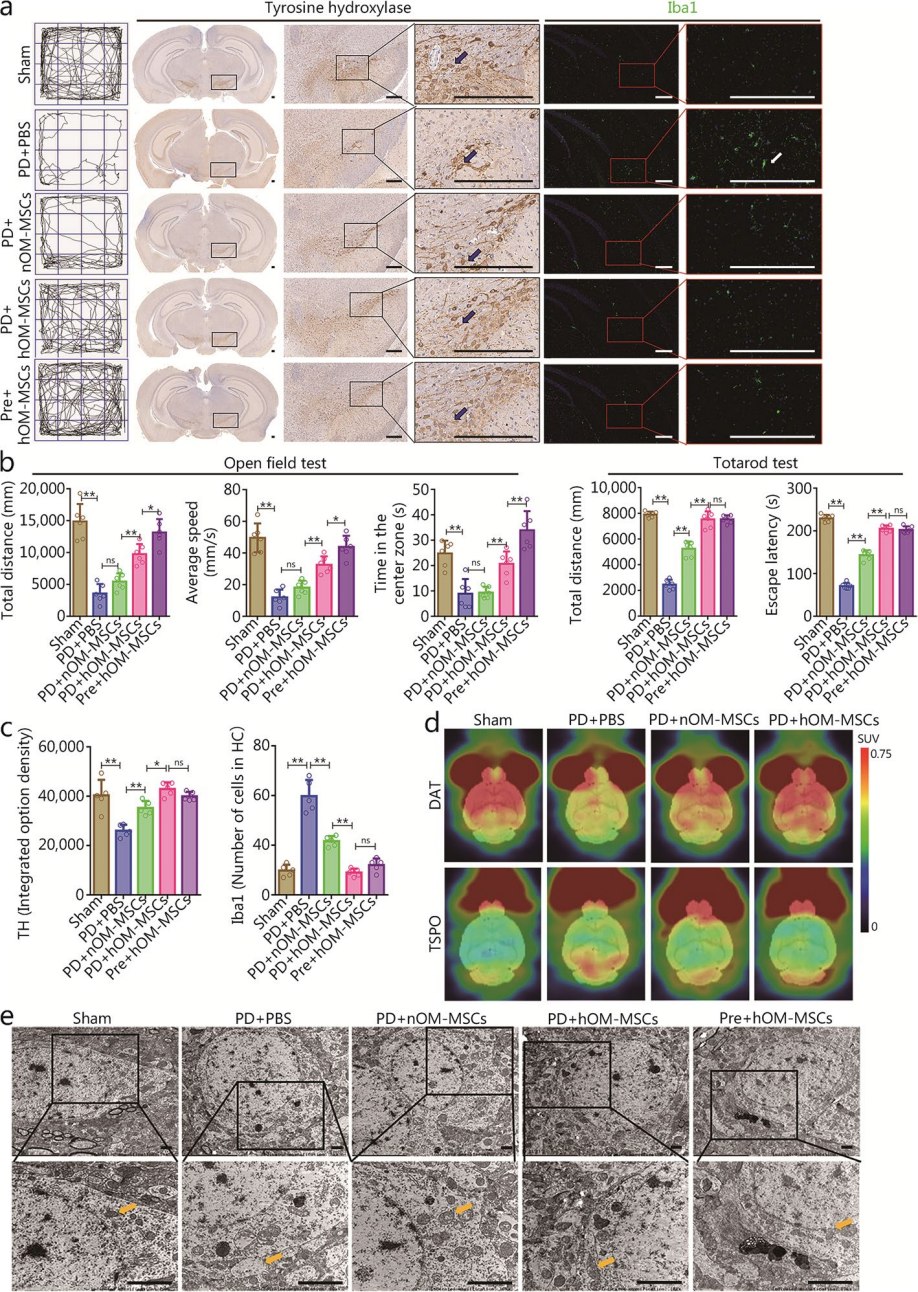
The hOM-MSCs facilitated the recovery of nerve function and the immunomodulation of microglia in the PD mouse model. **a** The neurologic function score of the open field test shows the activity trace of PD mice (left). The slide scanning technique shows TH^+^ cells (black arrow) immunohistochemical micrograph (medium), and shows Iba1^+^ cells (white arrow) immunofluorescence micrograph in the SN of PD mice (right) (scale bars = 200 μm). **b** The neurologic function score of the open field test shows the histogram of total distance, average speed, and central time, and the Tatarod test shows the histogram of total distance and escape latency (*n* = 6). **c** The histogram showing TH^+^ cell expression and the number of Iba1^+^ cells in (**a**). **d** CFT-PET brain imaging showing the distribution of DAT in brain tissue, and [^18^F]F-DPA brain imaging showing the distribution of activated microglia mitochondrial outer membrane TSPO in brain tissue. **e** TEM showing the mitochondria morphology and ultrastructure (yellow arrow) of neurons in the SN of PD mice (scale bars = 2 μm). Data are represented as mean ± SEM. ^*^P < 0.05, ^**^P < 0.01, ns non-significant. TH tyrosine hydroxylase, hOM-MSCs hypoxia-olfactory mucosa mesenchymal stem cells, SN substantia nigra, SUV standard uptake value, PD Parkinson’s disease, CFT 2-β-carbomethoxy-3β-(4-fluorophenyl)-(N-11C-methyl) tropan, PET positron emission tomography, DAT dopamine transporter, [^18^F]F-DPA N,N-diethyl-2-(2-(4-([^18^F]fluoro)phenyl)-5,7-dimethylpyrazolo[1,5-a]pyrimidin-3-yl)acetamide, TSPO transporter protein, TEM transmission electron microscope, PBS phosphate buffer saline

Open field and Tatarod tests were used to evaluate the neurologic function of the experimental groups. The results indicated a significant reduction in total distance, average speed, central time and escape latency in the PD + PBS group compared to the sham group. As compared to the PD + nOM-MSCs group, the PD + hOM-MSCs group and Pre + hOM-MSCs group showed the most substantial improvement in neural function (Fig. 2a, b). Therefore, transplantation of OM-MSCs enhanced neural function, whereas prevention and treatment with hOM-MSCs significantly improved neural function in a mouse model of PD.

The protective effect of hOM-MSCs on dopaminergic neurons in the SN of PD mice was assessed by immunohistochemistry and slide scanning to determine the loss of TH^+^ cells. The PD + PBS group showed a significant reduction in TH^+^ cells compared to the sham group, while treatment with nOM-MSCs increased TH^+^ cells. Notably, PD + hOM-MSCs and Pre + hOM-MSCs groups exhibited the most significant increase in TH^+^ cells. Meanwhile, immunostaining with Iba1, a marker for microglial activation, revealed a significant increase in the number of Iba1^+^ cells and pseudopodia in the PD + PBS group compared to the sham group. Furthermore, the number of Iba1^+^ cells decreased and cell morphology recovered in the PD + nOM-MSCs group. The PD + hOM-MSCs and Pre + hOM-MSCs groups also showed a significant decrease in the number of Iba1^+^ cells and a notable recovery of cell morphology (Fig. 2a, c). CFT-PET brain imaging was utilized to observe the distribution of DAT in brain tissue. The results demonstrated a significant reduction in the distribution of DAT within the SN, striatum, and hippocampus of the PD + PBS group compared to the sham group. Conversely, treatment with nOM-MSCs resulted in an increase in DAT distribution, while hOM-MSC treatment led to a significant increase as well (Fig. 2d). The distribution of activated microglia mitochondrial outer membrane transporter protein (TSPO) in brain tissue was demonstrated using [^18^F]F-DPA-PET brain imaging. TSPO distribution within the brain tissue was elevated in PD + PBS group compared to the sham group. However, treatment with nOM-MSCs resulted in reduced TSPO levels, while hOM-MSCs treatment exhibited significant reduction (Fig. 2d). The protein expression levels of BAX, Bcl-2, and caspase 3 were examined using Western blotting analysis on SN nerve cells. The results indicated that BAX and caspase 3 expression significantly increased whereas Bcl-2 expression decreased significantly when comparing PD model mice with control mice. Treatment with nOM-MSCs resulted in decreased levels of BAX and caspase 3 expression along with increased levels of Bcl-2 expression. Furthermore, both PD + hOM-MSCs and Pre + hOM-MSCs groups exhibited significantly reduced levels of BAX and caspase 3 expression along with elevated levels of Bcl-2 expression as well (Additional file 1: Fig. S4a). Additionally, the concentrations of DA and its metabolite HVA secreted by dopaminergic neurons in the SN were measured. ELISA results revealed a significant decrease in both DA and HVA concentrations in the PD + PBS group compared to the sham group. However, PD + nOM-MSCs group resulted in increased levels of both DA and HVA. Furthermore, both PD + hOM-MSCs and Pre + hOM-MSCs groups exhibited a significant elevation in DA and HVA concentrations (Additional file 1: Fig. S4b).

The flow cytometry analysis of JC-1 and ROS was conducted to serve as indicators of mitochondrial function, while alterations in mitochondrial ultrastructure were examined using TEM. The flow cytometry analysis revealed a significant decrease in JC-1 levels and an increase in ROS production in the PD + PBS group compared to the sham group. Conversely, treatment with nOM-MSCs resulted in increased JC-1 levels and decreased ROS production. The most pronounced increase in JC-1 levels and decrease in ROS production were observed in the PD + hOM-MSCs and Pre + hOM-MSCs groups (Additional file 1: Fig. S4c). TEM analysis demonstrated that the mitochondria from the PD + PBS group exhibited significant swelling and disorganization compared to those from the sham group. While nOM-MSCs improved mitochondrial morphology, hOM-MSCs showed the most remarkable recovery of mitochondrial structure (Fig. 2e). Thus, OM-MSCs exert a protective effect on neuronal mitochondrial function, with hOM-MSCs significantly enhancing this protective function.

Western blotting was employed to detect differences in levels of α-Syn, IL-1β, and CD206 protein expression. The findings revealed a significant elevation in α-Syn and IL-1β, as well as a notable reduction in CD206 within the PD + PBS group compared to the sham group. Conversely, following nOM-MSCs treatment, a decrease was observed in both α-Syn and IL-1β levels while CD206 levels increased. Furthermore, both α-Syn and IL-1β experienced a substantial decline alongside a significant increase in CD206 within the PD + hOM-MSCs and Pre + hOM-MSCs groups (Additional file 1: Fig. S4d). Therefore, the transplantation of hOM-MSCs significantly enhances the clearance of α-Syn and promotes the transformation to the M2 anti-inflammatory phenotype of microglia in PD mouse models. The protein expression levels of p50, p65, CD4^+^, and CD8^+^ in SN were evaluated using Western blotting. Notably, the PD + PBS group exhibited significant upregulation of p50, p65, CD4^+^, and CD8^+^ compared to the sham group. In contrast, the PD + nOM-MSCs group displayed a decrease in their expression levels, while both the PD + hOM-MSCs and Pre + hOM-MSCs groups demonstrated a substantial downregulation (Additional file 1: Fig. S4e). Thus, hOM-MSC transplantation effectively suppressed the inflammatory response and T-cell infiltration in the SN of the PD mouse model.

### ATAC-seq, scRNA-seq combined with ISO-seq were utilized to elucidate the underlying mechanisms of hOM-MSCs in ameliorating neural function impairment in PD models

**Fig. 3:**
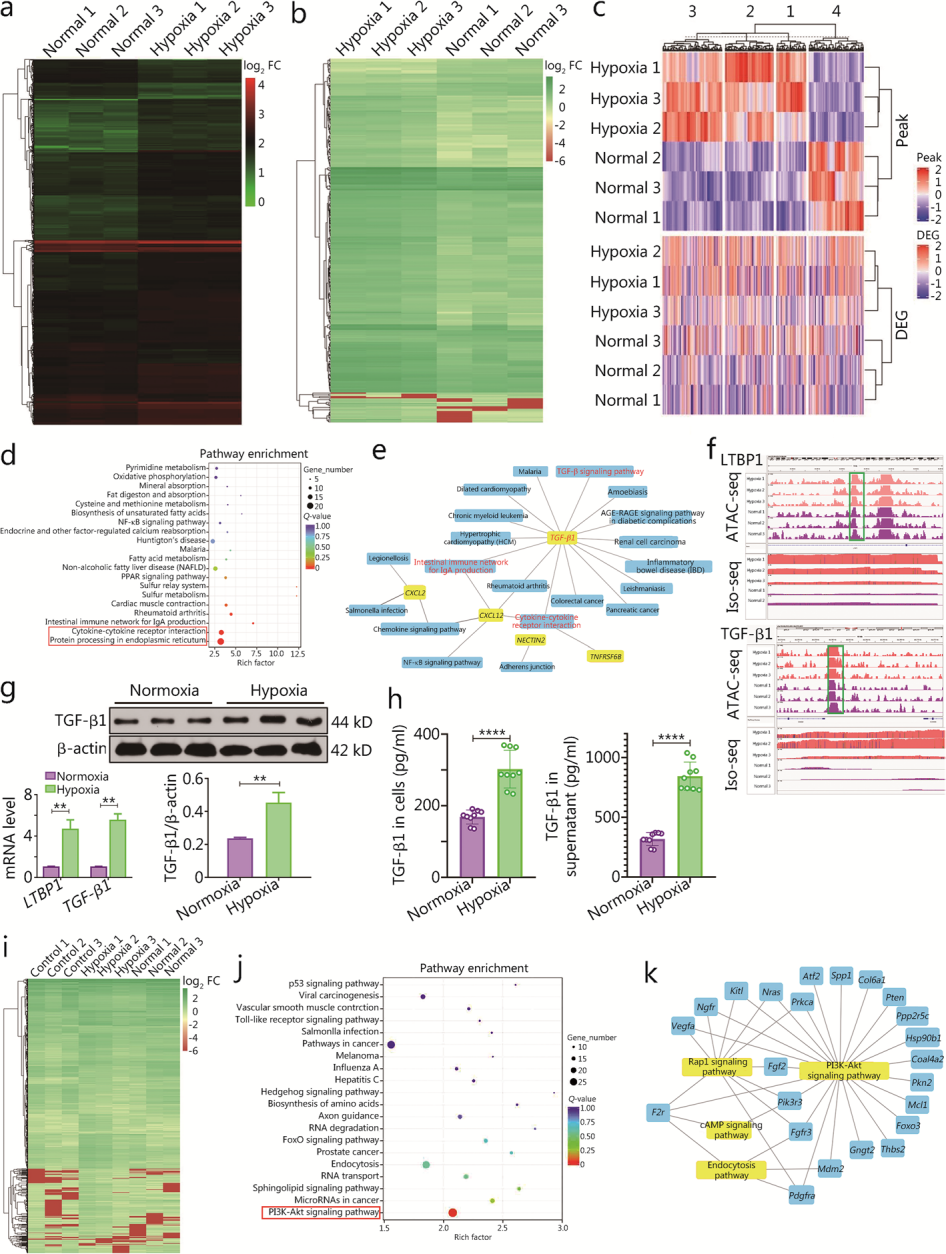
ATAC-seq combined with ISO-seq to elucidate the underlying mechanisms of hOM-MSCs in PD cell model. **a** Enrichment heatmap of ATAC-seq differential peak before and after hypoxic pretreatment of OM-MSCs. Each row is a differential peak, and each column is a sample. **b** Differential gene enrichment heatmap before and after hypoxic pretreatment of OM-MSCs. The horizontal coordinate represents the sample name and sample clustering results, and the vertical coordinate represents the differential genes and gene clustering results. **c** The intersection differential genes of ATAC-seq and ISO-seq are jointly displayed by clustering heatmap to visually display the dual changes of chromatin accessibility and expression level of genes. **d** Annotation results of DEGs were classified according to KEGG pathway types before and after hypoxic pretreatment of OM-MSCs. **e** The network relationships of the top 20 signaling pathways enriched by KEGG and their corresponding genes were mapped. **f** IGV visualized LTBP1 and TGF-β1 in the results of combined analysis of ATAC-seq and ISO-seq before and after hypoxic pretreatment of OM-MSCs. **g** RT-qPCR and Western blotting measuring the expression of *LTBP1* and *TGF-β1* mRNA and protein in OM-MSCs before and after hypoxia pretreatment. **h** ELISA showing a histogram of the concentration of TGF-β1 in OM-MSCs cells and supernatant both before and after hypoxia pretreatment. **i** Hierarchical cluster analysis was performed on the DEGs screened from microglia of the PD cell model, and the genes with the same or similar expression patterns were drawn into clustering heat maps. **j** Annotation results of DEGs were classified according to KEGG pathway types in microglia of the PD cell model after hOM-MSCs intervention. **k** The network relationships of the top 4 signaling pathways enriched by KEGG and their corresponding genes were mapped in the microglia of the PD cell model after hOM-MSCs intervention. ^**^P < 0.01, ^****^P < 0.0001. OM-MSCs olfactory mucosa mesenchymal stem cells, hOM-MSCs hypoxia-olfactory mucosa mesenchymal stem cells, KEGG Kyoto Encyclopedia of Genes and Genomes, TGF-β1 transforming growth factor-β1, LTBP1 latent transforming growth factor beta binding protein 1, DEGs differentially expressed genes, IGV integrative genomics viewer, ATAC-seq assay for transposase-accessible chromatin with high-throughput sequencing, ISO-seq isoform-sequencing, PD Parkinson’s disease, FC fold change, NF-κB nuclear factor kappa-B, PPAR peroxisome proliferator-activated receptor, FoxO forkhead box O

The immunomodulatory and neuroprotective mechanisms of hOM-MSCs in a PD cell model were determined using ATAC-seq combined with ISO-seq. Differences in the newly opened chromatin regions, corresponding transcripts, and related gene expression of hOM-MSCs were detected. The co-expression patterns of differentially regulated genes in ATAC-seq and ISO-seq datasets, both before and after hypoxic pretreatment of OM-MSCs, are visually depicted using a clustering heatmap to illustrate the concurrent changes in chromatin accessibility and gene expression levels (Fig. 3a-c). DeepTools, Boxplot, and Venn diagram were employed to visualize an ATAC-seq read enrichment heatmap for differential peaks, evaluate the heterogeneity of gene expression levels within a single sample, and depict co-occurring genes or specific changes in expression levels (Additional file 1: Fig. S5a-c). The annotation results of differentially expressed genes (DEGs) were categorized based on KEGG pathway types. Among them, the two signaling pathways that exhibited the most significant enrichment were cytokine-cytokine receptor interaction and protein processing in the endoplasmic reticulum (Fig. 3d). The DEGs of selected cytokine subclasses underwent hierarchical cluster analysis, and network interaction diagrams were elegantly crafted to showcase the 20 signaling pathways alongside their corresponding genes in the visually captivating KEGG enrichment bubble map. *TGF-β1* exhibited correlation with 15 signaling pathways, *CXCL12* correlated with 5 signaling pathways, *CXCL2* correlated with 3 signaling pathways, while the other two genes (*NECTIN2* and *TNFRSF6B*) only correlated with 1 signaling pathway. *CXCL12* and *CXCL2* were mainly involved in regulating cell chemotaxis, and *TGF-β1* had an anti-inflammatory, immunomodulatory role besides promoting cell differentiation (Fig. 3e). We hypothesized that hOM-MSCs secrete TGF-β1, a pivotal cytokine with immunomodulatory effects. Integrative genomics viewer (IGV) is a high-performance visualization tool for the interactive exploration of large, comprehensive genomic datasets that support various types of data. Both latent transforming growth factor beta binding protein 1 (LTBP1) and TGF-β1 were visualized by IGV using ATAC-seq combined with ISO-seq. The open chromatin region of LTBP1 in hOM-MSCs was located on chromatin 2, spanning from initiation site 33,288,817 to termination site 33,289,228, while the open chromatin region of TGF-β1 was found on chromatin 19, ranging from initiation site 44,259,010 to termination site 44,261,989. Furthermore, transcription coverage analysis revealed significantly increased expression levels of both LTBP1 and TGF-β1 in the hypoxia group (Fig. 3f). RT-qPCR quantification demonstrated a significant upregulation of *LTBP1* and *TGF-β1* mRNA levels in OM-MSCs under hypoxic conditions compared to the normoxia group (Fig. 3g). Western blotting analysis confirmed elevated protein expression levels of TGF-β1 in OM-MSCs within the hypoxia group when compared to the normoxia control (Fig. 3g). ELISA assessment before and after hypoxia pretreatment showed a substantial increase in the concentration of secreted TGF-β1 both intracellularly and extracellularly within the hypoxia group in contrast to the normoxia group; notably higher concentrations were observed in supernatant samples (Fig. 3h).

Furthermore, ISO-seq was performed on microglial cells derived from a PD cell model with the intervention of OM-MSCs. The control 1, control 2, and control 3 groups served as the control models. Normoxia 1, normoxia 2, and normoxia 3 underwent nOM-MSC intervention. Hypoxia 1, hypoxia 2, and hypoxia 3 received hOM-MSC intervention. The selected DEGs were subjected to hierarchical cluster analysis, and those with similar or identical expression patterns were visualized in clustering heat maps (Fig. 3i). Based on the KEGG pathway classification, the significantly enriched DEGs and transcripts between the model group and hOM-MSC intervention group were analyzed, and found that the phosphatidylinositol-3-kinase-protein kinase B (PI3K-Akt) signaling pathway was the most significant enrichment (Fig. 3j). In addition to identifying 4 highly enriched pathways along with their corresponding genes in the KEGG enrichment bubble map, our analysis revealed that there were 23 differentially enriched genes in the PI3K-Akt signaling pathway, 10 differentially enriched genes in the Rap1 signaling pathway, 4 differentially enriched genes in the endocytosis pathway, and only 2 distinctively enriched genes demonstrated in the cAMP signaling pathway (Fig. 3k). Hierarchical cluster analysis was conducted on the DEGs between the model and nOM-MSC intervention groups. Furthermore, KEGG pathway annotation revealed significant enrichment of DEGs between the two groups based on their respective pathways; however, no observed enrichment was found for the PI3K-Akt signaling pathway (Additional file 1: Fig. S5d). Hierarchical cluster analysis was also performed on the DEGs between the nOM-MSC and hOM-MSC groups while conducting a significant enrichment analysis based on KEGG pathway types for both DEGs and transcript annotations; revealing a higher level of enrichment for DEGs within the PI3K-Akt signaling pathway (Additional file 1: Fig. S5e). Thus, the PI3K-Akt signaling pathway is hypothesized to play a pivotal role in regulating microglial function.

**Fig. 4:**
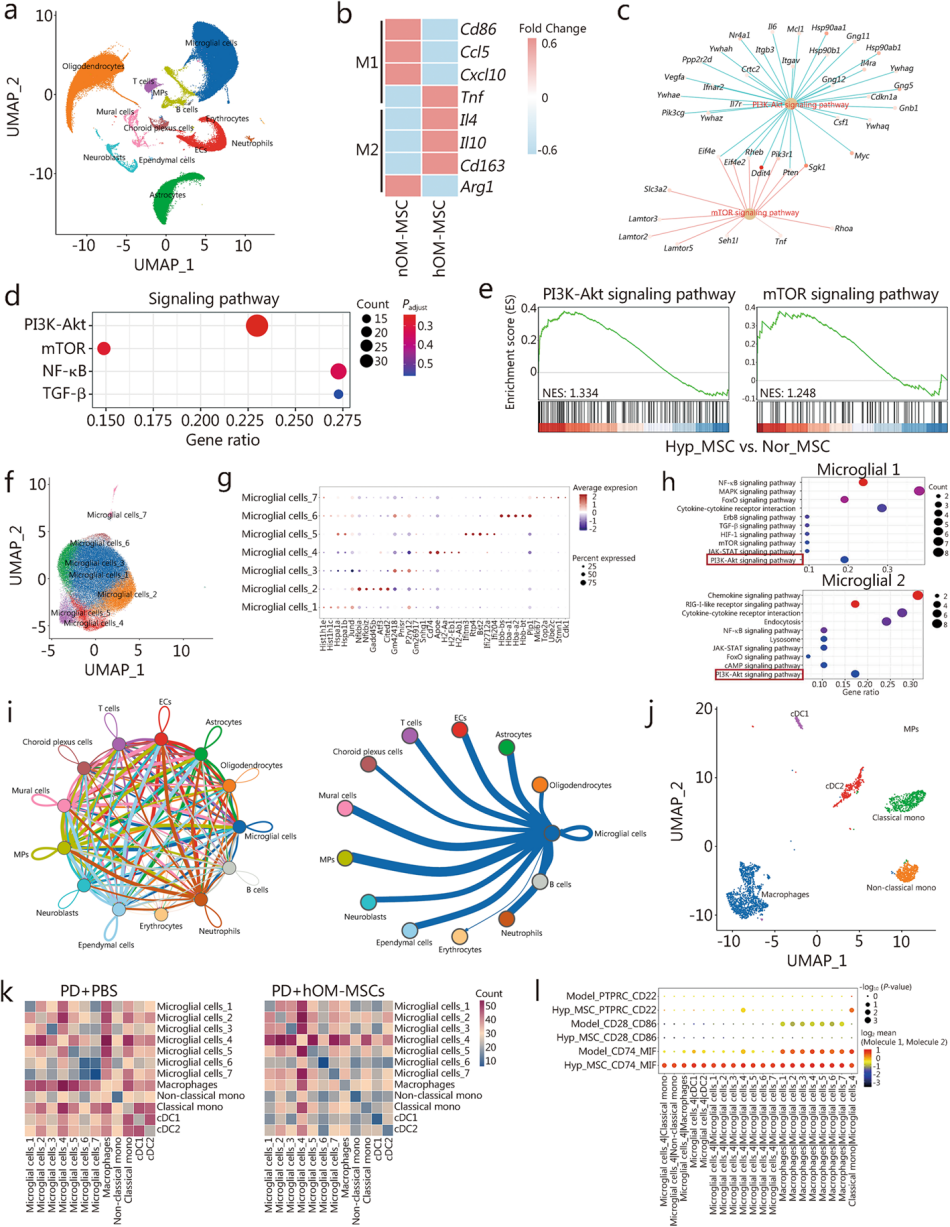
scRNA-seq to elucidate the underlying mechanisms of hOM-MSCs in PD mice model. **a** UMAP of integrated transcriptomes in all groups samples of SN showing cell-type assignment, including microglia cells, oligodendrocytes, astrocytes, ECs, MPs, neuroblasts, mural cells, choroid plexus cells, T cells, B cells, ependymal cells, erythrocytes, and neutrophils. **b** Heatmap showing microglial M1 markers and M2 markers between nOM-MSCs treatment and hOM-MSCs treatment in the SN of PD mice. **c** GSEA showing the two significantly enriched pathways and the corresponding genes in the enrichment bubble map. **d** Gene network analysis revealed significant enrichment and interaction of PI3K-Akt/mTOR signaling pathway in microglia. **e** Line plots of enrichment score showed upregulation of PI3K-Akt/mTOR signaling pathway in microglia. **f** UMAP of integrated transcriptomes in all groups of microglia showing cell-type assignment, and ultimately segregated the microglia into seven distinct subgroups. **g** Dotplot showing the genes expressed in > 10% of microglia in a cluster as DEGs. **h** KEGG pathway dotplot showing the enrichment and up-regulation signaling pathway in microglial cells_2 and _4 in the SN of PD mice after hOM-MSCs treatment. **i** The cell types net graph shows the interactions between various cell populations and pinpointed the interaction between microglia and MPs as the most significant. **j** UMAP of integrated transcriptomes in all groups of MPs showing cell-type assignment, including macrophages, non-classic and classic mononuclear cells, cDC1, and cDC2. **k** Heatmap showing the cell–cell interaction analysis based on known receptor-ligand interactions between all subtypes of microglia and MPs in the SN of PD mice after hOM-MSCs treatment. **l** Dot graph showing the cell–cell interaction differences among the three pairs of receptor-ligands were the most significant. PI3K phosphoinositide 3-kinase, Akt protein kinase B, MPs mononuclear phagocytes, ECs endothelial cells, TGF-β transforming growth factor-β, mTOR mammalian target of rapamycin, hOM-MSCs hypoxia-olfactory mucosa mesenchymal stem cells, nOM-MSCs normoxia-olfactory mucosa mesenchymal stem cells, PD Parkinson’s disease, UMAP uniform manifold approximation and projection, SN substantia nigra, GSEA Gene Set Enrichment Analysis, KEGG Kyoto Encyclopedia of Genes and Genomes, NES normalized enrichment score, MAPK mitogen-activated protein kinase, FoxO forkhead box O, ErbB receptor tyrosine kinases, HIF-1 hypoxia inducible factor-1, JAK janus tyrosine kinase, STAT signal transducer and activator of transcription, mono mononuclear

To elucidate the immunomodulatory and neuroprotective mechanisms of hOM-MSCs in a mouse model of PD, scRNA-seq was used to identify gene expression profiles in SN tissue obtained from the transplantation of hOM-MSCs in the PD mouse model. Utilizing the uniform manifold approximation and projection (UMAP) algorithm allowed us to visualize different cell types across all groups within a two-dimensional space, including microglia cells, oligodendrocytes, astrocytes, endothelial cells (ECs), mononuclear phagocytes (MPs), neuroblasts, mural cells, choroid plexus cells, T cells, B cells, ependymal cells, erythrocytes, and neutrophils (Fig. 4a). To identify DEGs, the genes that were expressed in more than 10% of the cells within a cluster were selected with an average log (fold change) > 0.25 as DEGs (Additional file 1: Fig. S5f). The microglia cells, which constitute the predominant population and play a crucial role in the pathogenesis and progression of neuroinflammatory responses in PD. M1 markers *(Cd86*, *Ccl5*, *Cxcl10*, and* Tnf*) and M2 markers (*Il4*, *Il10*, *Cd163*, and *Arg1*) were compared in nOM-MSC treatment, hOM-MSC treatment and prevention. The microglia in the hOM-MSC and Pre-hOM-MSC groups showed a stronger inclination towards M2 transformation compared to the nOM-MSC treatment group (Fig. 4b; Additional file 1: Fig. S5g). Furthermore, we conducted a Gene Set Enrichment Analysis to compare and analyze the microglial gene sets between the hOM-MSC and Pre-hOM-MSC groups and the nOM-MSC group. An enrichment bubble map identified two significantly enriched pathways with their corresponding genes (Fig. 4c; Additional file 1: Fig. S5h). Gene network analysis revealed significant enrichment and interaction of PI3K-Akt/mammalian target of rapamycin (mTOR) signaling pathway in microglia (Fig. 4d; Additional file 1: Fig. S5i). Line plots of enrichment scores demonstrated upregulation of the PI3K-Akt/mTOR signaling pathway in microglia when comparing the hOM-MSC and Pre-hOM-MSC groups with the nOM-MSC group (Fig. 4e; Additional file 1: Fig. S5j). Therefore, based on our further analysis, the PI3K-Akt/mTOR signaling pathway has a pivotal role in microglia during hOM-MSC treatment and prevention in the PD mouse model. Therefore, based on our further analysis, the PI3K-Akt/mTOR signaling pathway has a pivotal role in microglia during hOM-MSC treatment and prevention in the PD mouse model.

After painstakingly categorizing the microglia cells into detailed subtypes, they were further segregated into 7 distinct subgroups. And then selected the genes expressed in more than 10% of cells in a cluster as DEGs (Fig. 4f, g). Enrichment analysis of KEGG was conducted on microglia subsets across different groups. Comparing the PD + hOM-MSCs and Pre-hOM-MSCs groups with the PD + PBS group, significant enrichment in the PI3K-Akt signaling pathway was observed for microglial cells_2 and _4 (Fig. 4h; Additional file 1: Fig. S5k). The interaction between microglia and MPs was particularly noteworthy among the various cell populations investigated (Fig. 4i). Using the UMAP algorithm to visualize the captured cell subtype of MPs in all groups within a two-dimensional space, including macrophages, non-classic and classic mononuclear cells, cDC1, and cDC2 (Fig. 4j). The cell–cell interaction analysis was performed based on known receptor-ligand interactions between all subtypes of microglia and MPs. Our analysis revealed that the PD + hOM-MSCs and Pre-hOM-MSCs groups exhibited significantly enhanced cell–cell interactions between microglial cells_4 and all other cell subtypes compared to the PD + PBS group (Fig. 4k; Additional file 1: Fig. S5l). The differences in cell–cell interactions among three pairs of receptor-ligands were found to be most significant when comparing PD + hOM-MSCs group with the PD + PBS group, including protein tyrosine phosphatase receptor type C (PTPRC) (classic mononuclear cells)-CD22 (microglial cells_4), CD28 (macrophages)-CD86 (microglia) and CD74 (microglial cells_4)-macrophage migration inhibitory factor (MIF) (MPs) (Fig. 4l). The same approach analysis was performed to compare the Pre-hOM-MSCs group with the PD + PBS group. Notably, significant differences were observed among the three pairs of receptor-ligands, including IL1B (MPs)-adrenoceptor beta 2 (ADRB2) (microglia), G protein-coupled receptor 37 (GPR37) (MPs)-prosaposin (PSAP) (microglial cells_4) and CD74 (MPs)-MIF (microglia) (Additional file 1: Fig. S5m). Therefore, the PI3K-Akt signaling pathway plays a crucial role in microglial cells_2 and _4 during hOM-MSC treatment and prevention in the PD mouse model. Moreover, interactions between PTPRC-CD22, CD28-CD86, CD74-MIF, IL1B-ADRB2, and GPR37-PSAP receptor-ligand may significantly impact the regulation of immunity and phagocytosis in microglial cells. The same approach analysis was performed between the Pre-hOM-MSC group and PD + PBS group.

### TGF-β1 mediates hOM-MSCs to enhance neuroprotective function by activating the PI3K-Akt signaling pathway in microglia in vitro

After conducting high-throughput sequencing analysis, the differential gene enrichment in the PI3K-Akt pathway diagram of microglia cells was compared subsequent to hOM-MSC intervention. Our findings suggested that growth factor located extracellularly activates the PI3K-Akt signaling pathways by binding to receptor tyrosine kinase (RTK; Additional file 1: Fig. S6a). Furthermore, TGF-β1 is a protein factor present in growth factors, and anaplastic lymphoma kinase (ALK) is an RTK belonging to the type I receptor of TGF-β1. Based on the aforementioned analysis, we propose that hOM-MSC-secreted TGF-β1 may modulate immune responses by activating the PI3K-Akt signaling pathway through binding to microglial cell membrane receptors expressing ALK.

**Fig. 5:**
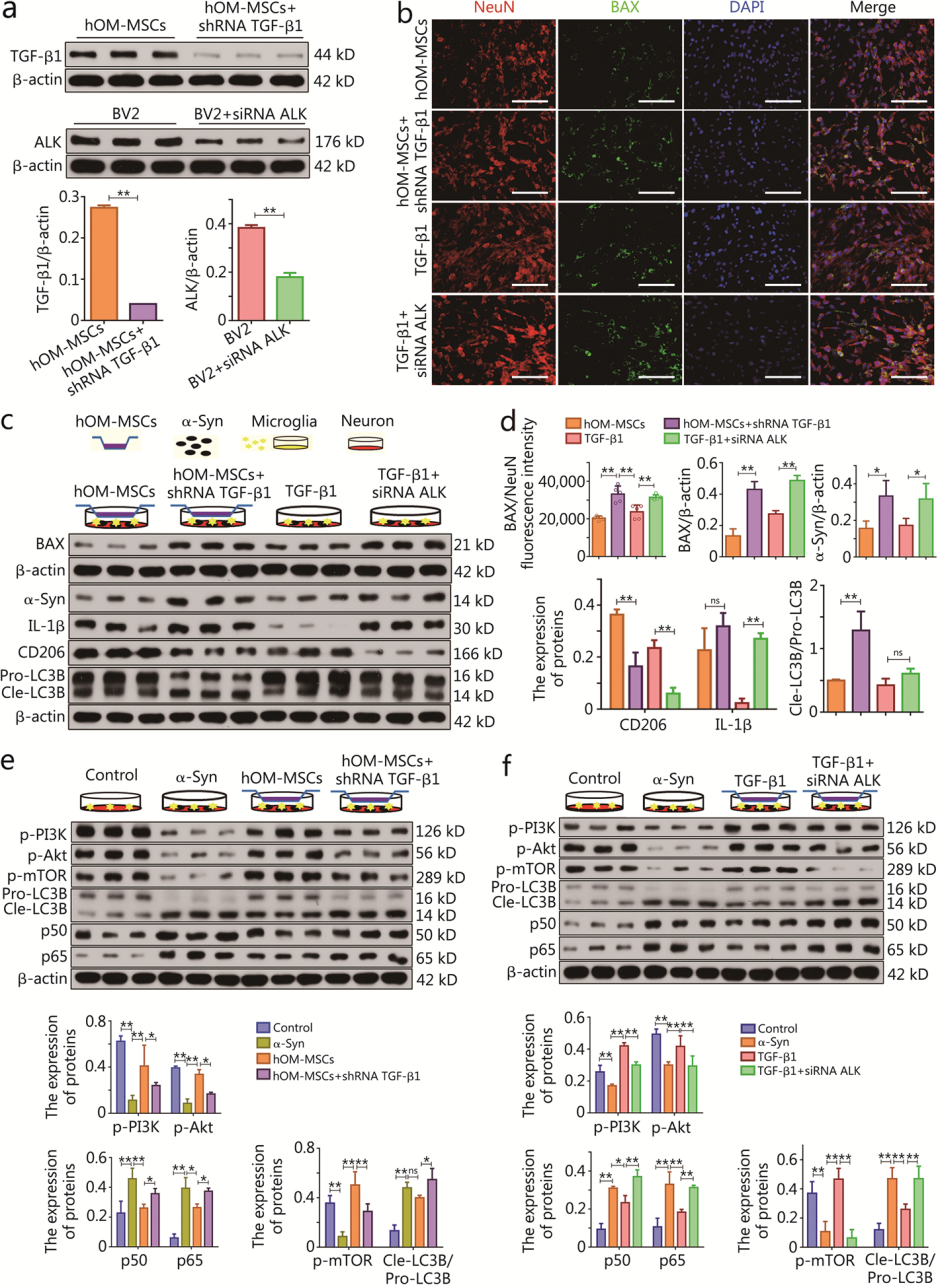
TGF-β1 mediates hOM-MSC to enhance neuroprotective function by activating the PI3K-Akt signaling pathway in microglia in vitro*.* **a** Western blotting measuring TGF-β1 protein expression in hOM-MSCs, and ALK protein expression in BV2 cells. **b** Exemplary immunofluorescence micrograph showing nuclei (DAPI), NeuN, and BAX expression in SH-SY5Y cells (Scale bars = 40 μm). **c** Western blotting measuring BAX protein expression in SH-SY5Y cells, and α-Syn, IL-1β, CD206, and LC3B protein expression in BV2 cells. **d** The histogram showing the BAX fluorescence expression in (**b**), and BAX, α-Syn, IL-1β, CD206, and LC3B protein expression in (**c**). **e**,** f** Western blotting measuring p-PI3K, p-Akt, p-mTOR, p50, p65, and LC3B protein expression in BV2 cells. Data are represented as mean ± SEM. ^*^P < 0.05, ^**^P < 0.01, ns non-significant. hOM-MSCs hypoxia-olfactory mucosa mesenchymal stem cells, TGF-β1 transforming growth factor-β1, ALK anaplastic lymphoma kinase, NeuN neuron-specific nuclear protein, DAPI 4’,6-diamidino-2-phenylindole, α-Syn α-synuclein, IL-1β interleukin-1β, PI3K phosphoinositide 3-kinase, Akt protein kinase B, mTOR mammalian target of rapamycin

The lentiviral vectors were employed to generate shRNA specifically targeting *TGF-β1* mRNA in hOM-MSCs, while siRNA against the cell surface receptor, ALK, was utilized in BV2 microglial cells. The Western blotting results demonstrated the remarkable efficacy of the two mRNA interference reagents constructed, which can be employed for subsequent functional verification experiments (Fig. 5a). The neuroprotective effect of hOM-MSCs mediated by TGF-β1 on neurons in PD cell models was investigated, along with the ability of microglia to regulate immune responses and clear α-Syn. Through dual staining of the neuronal marker NeuN and the apoptotic marker BAX, an increase in apoptotic cells was observed in the hOM-MSCs + shRNA TGF-β1 group compared to the hOM-MSCs group. Conversely, a significant decrease in apoptotic cells was noted in the TGF-β1 group, while a notable increase in apoptotic cells was observed in the TGF-β1 + siRNA ALK group (Fig. 5b). Furthermore, Western blotting analysis of BAX protein expression provided additional evidence supporting the protective effect of hOM-MSCs mediated by TGF-β1 on neurons in PD cell models (Fig. 5c, d). The levels of α-Syn, M1 marker (IL-1β), M2 marker (CD206), and autophagy-related LC3B expression were further assessed in microglia from each group. Western blotting analysis revealed that the hOM-MSCs + shRNA TGF-β1 group exhibited upregulated expression of α-Syn, IL-1β, and LC3B protein compared to the hOM-MSCs group, while CD206 protein expression was significantly downregulated. Conversely, the TGF-β1 group showed decreased expression of α-Syn, IL-1β, and LC3B, but increased CD206 expression. The levels of α-Syn, IL-1β, and LC3B were significantly upregulated in the TGF-β1 + siRNA ALK group, whereas CD206 expression was markedly downregulated (Fig. 5c, d). To assess the regulatory impact of TGF-β1-mediated hOM-MSCs on inflammatory factors in the PD cell model, the levels of proinflammatory cytokines (IL-1β and TNF-α) and anti-inflammatory cytokines (IL-4 and IL-10) were assessed in the cell supernatant of each experimental group. ELISA results revealed that compared to the hOM-MSCs group, there was a significant increase in concentrations of IL-1β and TNF-α in the supernatant of the hOM-MSCs + shRNA TGF-β1 group, while concentrations of IL-4 and IL-10 were significantly reduced. Furthermore, there was a notable decrease in levels of IL-1β and TNF-α in the supernatants of the TGF-β1 group, accompanied by a significant elevation in concentrations of IL-4 and IL-10. The concentrations of IL-1β and TNF-α were markedly increased in the TGF-β1 + siRNA ALK group, whereas levels of IL-4 and IL-10 were substantially decreased (Additional file 1: Fig. S6b). Therefore, TGF-β1 mediates the ability of hOM-MSCs to regulate the immunophenotype, autophagy, and clearance of α-Syn in microglia. Furthermore, the administration of human recombinant protein TGF-β1 alone is also capable of fulfilling these functions.

Immunologic regulation by hOM-MSCs via activation of the PI3K-Akt signaling pathway in microglia cell secretion of TGF-β1 was determined. The phosphorylation status of the PI3K-Akt signaling pathway, the mTOR-mediated regulation of cellular activity and autophagic homeostasis, as well as the levels of nuclear factor kappa-B (NF-κB) family member (p50 and p65) and LC3B autophagy-related proteins involved in immune-inflammatory responses, were assessed in the experimental groups. Western blotting results revealed that the α-Syn group exhibited a significant decrease in the phosphorylation levels of PI3K, Akt, and mTOR compared to the control group, while p50, p65, and LC3B were significantly upregulated. Conversely, hOM-MSC intervention led to a significant increase in phosphorylation levels of PI3K, Akt, and mTOR with concomitant downregulation of p50, p65, and LC3B. In contrast, hOM-MSCs + shRNA TGF-β1 intervention resulted in inhibited phosphorylation levels of PI3K, Akt, and mTOR, while p50, p65, and LC3B were activated (Fig. 5e). The involvement of human recombinant protein TGF-β1 in immune regulation was investigated in microglial cells by examining its impact on the activation of the PI3K-Akt signaling pathway. The phosphorylation status of the PI3K-Akt and mTOR signaling pathways, as well as the levels of LC3B, p50, and p65 proteins, were assessed in these experimental groups. Western blotting results revealed that compared to the control group, the α-Syn group exhibited a significant decrease in the phosphorylation levels of PI3K, Akt, and mTOR while showing significant upregulation of p50, p65, and LC3B. Conversely, TGF-β1 intervention led to a significant increase in the phosphorylation levels of PI3K, Akt, and mTOR with concomitant downregulation of p50, p65, and LC3B. In contrast, TGF-β1 + siRNA ALK intervention resulted in inhibited phosphorylation levels of PI3K, Akt, and mTOR, and activated p50, p65, and LC3B (Fig. 5f). Therefore, the secretion of TGF-β1 by hOM-MSCs further activated the PI3K-Akt signaling pathway through interaction with microglial cell membrane ALK receptors, thereby regulating the immune response and maintaining homeostasis of autophagy.

### TGF-β1 mediates hOM-MSCs to facilitate the recovery of nerve function by activating the PI3K-Akt signaling pathway in microglia in vivo

The ability of hOM-MSC transplantation to secrete TGF-β1 and activate the PI3K-Akt signaling pathway by binding with ALK-expressing microglial cell membrane receptors was further confirmed, thereby exerting regulatory immune response and neuroprotective functions in PD mouse models. Lentiviral vectors were employed to generate shRNA targeting *TGF-β1* mRNA in hOM-MSCs, while AAV was used to produce AAV ALK targeting the microglial cell surface receptor, ALK, in a PD mouse model.

**Fig. 6:**
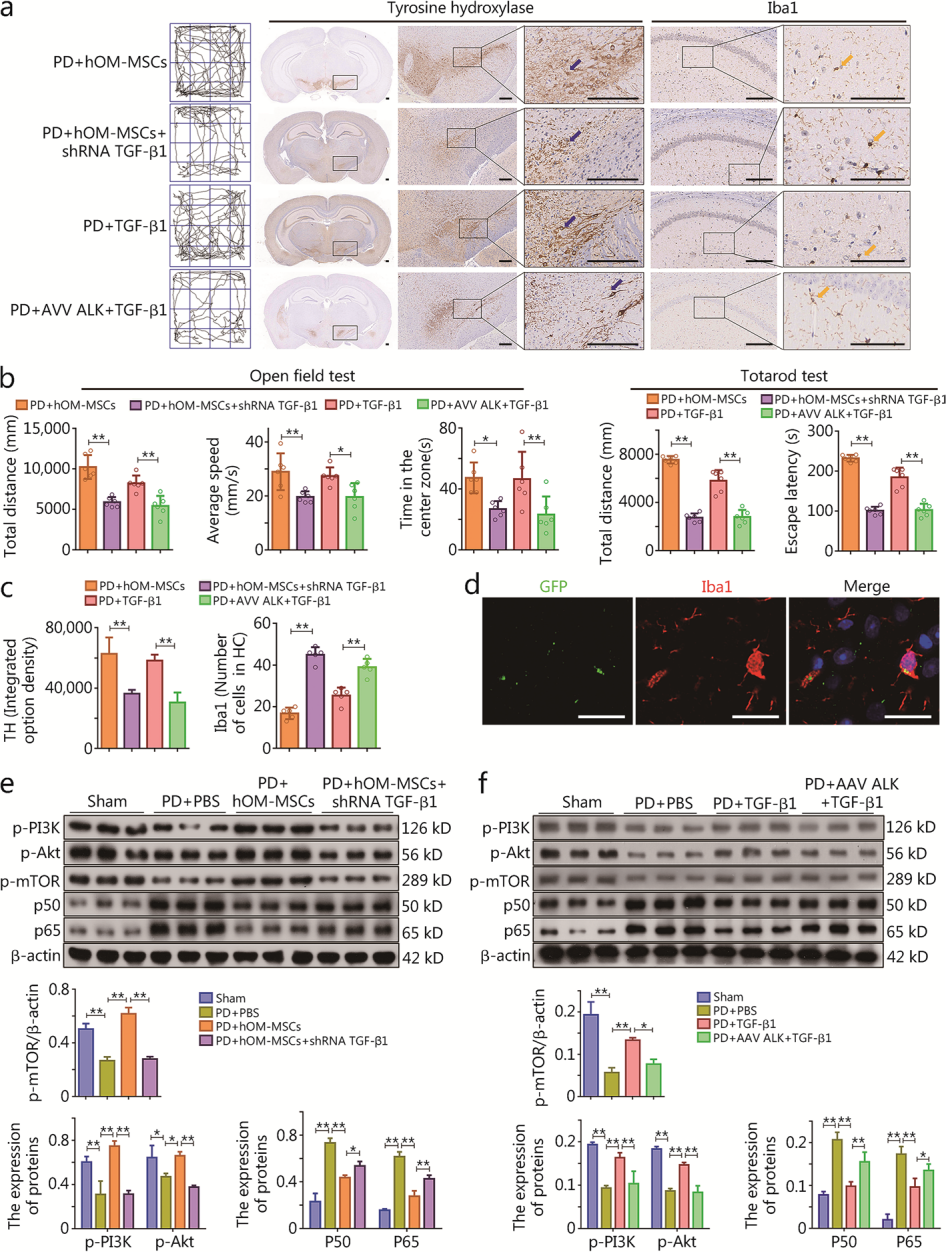
TGF-β1 mediates hOM-MSC to facilitate the recovery of nerve function by activating the PI3K-Akt signaling pathway in microglia in vivo. **a** The neurologic function score of open field test showing the activity trace of PD mice (left). The slide scanning technique shows TH^+^ cells immunohistochemical micrograph (medium) and shows Iba1^+^ cells immunohistochemical micrograph in the SN of PD mice (right) (scale bars = 200 μm). **b** The neurologic function score of the open field test shows the histogram of total distance, average speed, and central time (*n* = 6), and the Tatarod test shows the histogram of total distance and escape latency (*n* = 6). **c** The histogram showing TH^+^ cell expression and the number of Iba1^+^ cells in (**a**). **d** Exemplary immunofluorescence micrograph showing nuclei (DAPI), AAV ALK-GFP, and Iba1 co-localization expression in microglia of SN in PD mice (scale bars = 20 μm). **e**,** f** Western blotting measuring p-PI3K, p-Akt, p-mTOR, p50, and p65 protein expression in SN of PD mice. Data are represented as mean ± SEM. ^*^P < 0.05, ^**^P < 0.01. TH tyrosine hydroxylase, GFP green fluorescent protein, PI3K Phosphoinositide 3-kinase, Akt protein kinase B, mTOR mammalian target of rapamycin, hOM-MSCs hypoxia-olfactory mucosa mesenchymal stem cells, PD Parkinson’s disease, TGF-β1 transforming growth factor-β1, ALK anaplastic lymphoma kinase, AAV adeno-associated virus

The therapeutic potential of TGF-β1 in a mouse model of PD was investigated by examining its binding to the ALK receptor on microglial cell membranes, followed by modulation of hOM-MSCs. The neurologic function of the experimental groups was evaluated using the open field and Tatarod tests. The findings demonstrated a noteworthy decrease in total distance, average speed, central time, and latency within the PD + hOM-MSCs + shRNA TGF-β1 group compared to the PD + hOM-MSCs group, these aforementioned indices of neurologic function exhibited a significant increase in the PD + TGF-β1 group, whereas there was a marked decrease when comparing the PD + AAV ALK + TGF-β1 group with the PD + TGF-β1 group (Fig. 6a, b). After immunohistochemical staining with the dopaminergic neuron marker, TH, slide scanning was performed to observe the loss of TH^+^ cells in the SN. The findings revealed a significant decrease in the proportion of TH^+^ cell loss in the PD + hOM-MSCs + shRNA TGF-β1 group compared to the PD + hOM-MSCs group. Treatment with TGF-β1 substantially mitigated this proportion, while administration of AAV ALK + TGF-β1 also resulted in a decrease (Fig. 6a, c). After immunohistochemical staining with the microglia activation marker, Iba1, slide scanning was performed to observe the loss of Iba1^+^ cells in the hippocampus. The results demonstrated a significant increase in the proportion of Iba1^+^ cells in the PD + hOM-MSCs + shRNA TGF-β1 group compared to the PD + hOM-MSCs group. Treatment with TGF-β1 significantly reduced the proportion of Iba1^+^ cells, and treatment with AAV ALK + TGF-β1 also showed an increase in this proportion (Fig. 6a, c). Immunofluorescence co-localization results demonstrated the precise targeting ability of AAV ALK towards microglial cell membrane receptors in the SN of a PD mouse model (Fig. 6d). The regulatory impact of TGF-β1-mediated hOM-MSCs on inflammatory factors in the SN of the PD mouse model was evaluated by assessing the levels of both proinflammatory cytokines (IL-1β and TNF-α) and anti-inflammatory cytokines (IL-4 and IL-10) in the SN tissues from each experimental group. The ELISA results unveiled a significant elevation in the concentrations of IL-1β and TNF-α within the PD + hOM-MSCs + shRNA TGF-β1 group compared to PD + hOM-MSCs group. Conversely, there was a marked reduction in IL-4 and IL-10 levels. Furthermore, TGF-β1 treatment resulted in a noteworthy decrease in IL-1β and TNF-α levels, while significantly elevating the concentrations of IL-4 and IL-10. On the other hand, administration of AAV ALK along with TGF-β1 led to an augmentation in both IL-1β and TNF-a levels, accompanied by a substantial reduction in the concentrations of IL-4 and IL-10 (Additional file 1: Fig. S6c). Therefore, TGF-β1 mediates the modulatory effects of hOM-MSCs on microglia immunophenotype, and human recombinant protein TGF-β1 alone is also capable of fulfilling these roles.

**Fig. 7:**
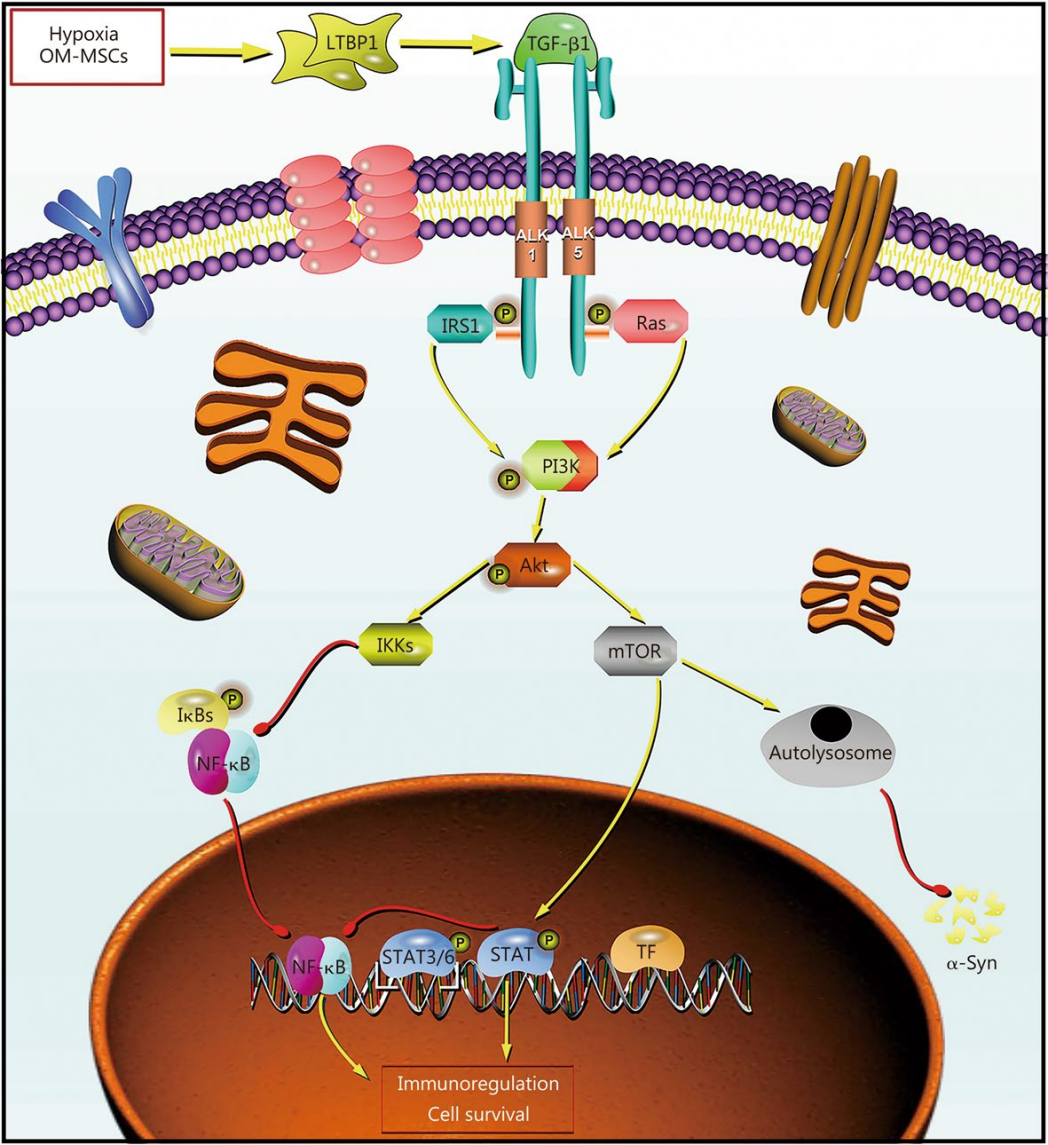
Graphical mechanism showing the secretion of TGF-β1 by hOM-MSCs further activates the PI3K-Akt signaling pathway through interaction with microglial cell membrane ALK receptors, thereby regulating the immune response and maintaining autophagy homeostasis. OM-MSCs olfactory mucosa mesenchymal stem cells, LTBP1 latent transforming growth factor beta binding protein 1, TGF-β1 transforming growth factor-β1, ALK anaplastic lymphoma kinase, IRS1 insulin receptor substrate 1, Ras rat sarcoma, PI3K Phosphoinositide 3-kinase, Akt Protein protein kinase B, mTOR mammalian target of rapamycin, IKKs The IκB kinase, IκBs inhibitory κB, NF-κB nuclear factor kappa-B, STAT signal transducer and activator of transcription, TF transcription factor

The immunologic regulation of hOM-MSCs through activation of the PI3K-Akt signaling pathway was further investigated in microglia cells that secret TGF-β1. Western blotting results revealed a significant decrease in phosphorylation levels of PI3K, Akt, and mTOR in the PD + PBS group compared to the sham group; however, there was a significant upregulation of p50 and p65. Conversely, hOM-MSC treatment led to a significant increase in phosphorylation levels of PI3K, Akt, and mTOR along with concomitant downregulation of p50 and p65. In contrast, treatment with hOM-MSCs + shRNA TGF-β1 resulted in inhibited phosphorylation levels of PI3K, Akt, and mTOR, as well as activation of p50 and p65 (Fig. 6e). Western blotting results unveiled a remarkable reduction in the phosphorylation levels of PI3K, Akt, and mTOR in the PD + PBS group compared to the sham group, while simultaneously exhibiting a significant upregulation of p50 and p65. Conversely, treatment with TGF-β1 led to a significant increase in phosphorylation levels of PI3K, Akt, and mTOR along with concomitant downregulation of p50 and p65. In contrast, AAV ALK + TGF-β1 treatment inhibited phosphorylation levels of PI3K, Akt, and mTOR, as well as the activation of p50 and p65 (Fig. 6f). Therefore, the secretion of TGF-β1 by hOM-MSCs further activates the PI3K-Akt signaling pathway through interaction with microglial cell membrane ALK receptors, thereby regulating the immune response (Fig. 7).

### Phase I hOM-MSC implantation clinical trial for PD patients

**Fig. 8:**
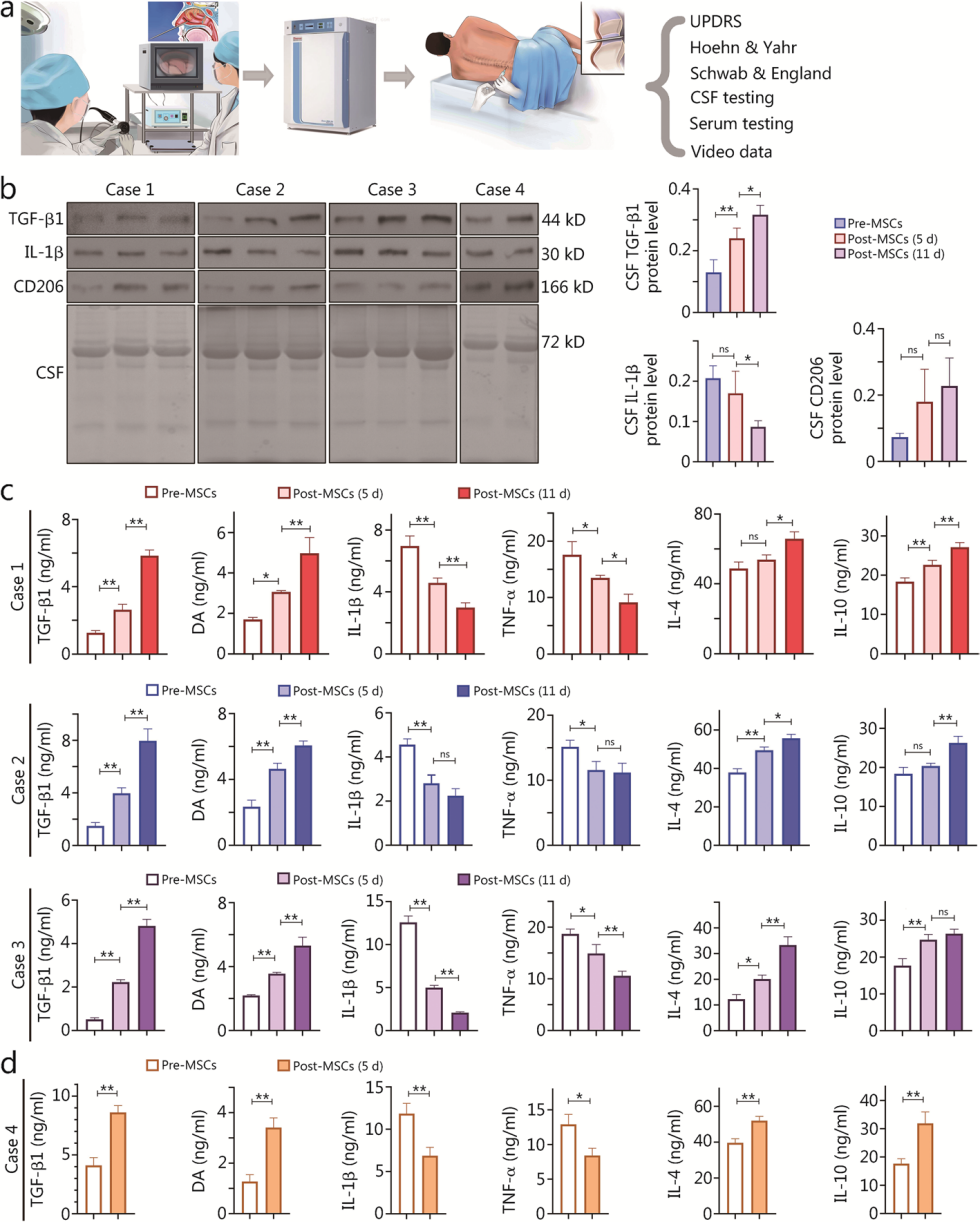
Phase I hOM-MSC implantation clinical trial for PD patients. **a** The strategy for hOM-MSCs transplants in PD patients. The process is divided into the collection of patient nasal mucosa, cultivation of olfactory mucosa mesenchymal stem cells, lumbar puncture transplantation of cells, and evaluation of patients and collected specimens.** b** Western blotting measuring TGF-β1, IL-1β, and CD206 protein expression in CSF of PD patients. **c** ELISA showing histogram of TGF-β1, DA, IL-1β, TNF-α, IL-4, and IL-10 protein concentration in CSF of case 1/2/3 before hOM-MSCs transplantation as well as 5 and 11 d after transplantation. **d** ELISA showing a histogram of TGF-β1, DA, IL-1β, TNF-α, IL-4, and IL-10 protein concentration in CSF of case 4 before hOM-MSCs transplantation and 5 d after transplantation. Data are represented as mean ± SEM. ^*^P < 0.05, ^**^P < 0.01, ns non-significant. UPDRS unified Parkinson’s disease rating scale, CSF cerebrospinal fluid, TGF-β1 transforming growth factor-β1, IL-1β interleukin-1β, DA dopamine, TNF-α tumor necrosis factor-α, IL-4 interleukin-4, IL-10 interleukin-10, Pre-MSCs previous treatment olfactory mucosa mesenchymal stem cells, Post-MSCs post-treatment olfactory mucosa mesenchymal stem cells

A total of 5 patients underwent intraspinal implantation of hOM-MSCs, followed by serum and cerebrospinal fluid analysis post-transplantation, with a subsequent follow-up period ranging from 1 to 6 months (Fig. 8a; Additional file 2: Table S1). The procedure was well-tolerated by all subjects, and no severe adverse events were reported during the post-procedure period (Fig. 8a).

The first participant in the study was a 67-year-old female (case 1) who had been suffering from PD for two decades. Following the implantation of hOM-MSCs, there was a significant reduction in the oral maintenance dose of levodopa, decreasing from 1.0 g/d before surgery to 0.5 g/d. The patient exhibited improvements in mood, activities of daily living, motor tests, and treatment complications (Additional file 3: Video S1). One month after treatment, the total UPDRS score decreased from 79 to 51. Additionally, improvement was observed on both the Hoehn and Yahr rating scale (from level 3 to level 2) and the Schwab and England daily activity scale (from 50% recovery to 70%). The results obtained during the 6-month follow-up revealed a UPDRS total score of 69, a Hoehn and Yahr rating scale at level 2.5, and a Schwab and England daily activity scale at 60% (Additional file 2: Table S2). Case 1 exhibited good tolerance to the procedure, with no reported serious adverse events during the post-procedure period.

The second participant in the cohort was a 79-year-old male (case 2) who was diagnosed with PD for a duration of 10 years. After implantation of hOM-MSCs, the oral maintenance dose of levodopa was significantly reduced from 0.75 g/d prior to surgery to 0.25 g/d. The patient demonstrated enhancements in affect, instrumental activities of daily living, and motor function assessments (Additional file 4: Video S2). One month after treatment, the total UPDRS score decreased from 89 to 61. Additionally, there was an improvement observed in the Hoehn and Yahr rating scale from level 3 to level 2. An enhancement was noted in the Schwab and England daily activity scale which showed recovery from 50 to 70%. The 6-month follow-up results post-surgery revealed a UPDRS total score of 78, Hoehn and Yahr rating scale of level 3, and Schwab and England daily activity scale of 60% (Additional file 2: Table S3). Case 2 demonstrated excellent tolerance to this procedure with no serious adverse events reported during the post-procedure period.

The third participant in the cohort of this phase I trial was a 67-year-old male (case 3) who had been diagnosed with PD for two decades. After hOM-MSCs were implanted, the oral maintenance dose of levodopa experienced a significant reduction from 1.0 g/d before surgery to 0.25 g/d. The patient exhibited notable improvements in mood, activities of daily living, motor tests, and treatment complications (Additional file 5: Video S3). One month after treatment, the total UPDRS score decreased from 85 to 59. Additionally, an improvement was observed in the Hoehn and Yahr rating scale from level 3 to level 2. The Schwab and England daily activity scale showed recovery from 50 to 70%. The 6-month follow-up results post-surgery showed a UPDRS total score of 80, a Hoehn and Yahr rating scale at level 3, and a Schwab and England daily activity scale at 50% (Additional file 2: Table S4). Case 3 demonstrated excellent tolerance to the procedure with no severe adverse events reported during the post-procedure period.

The fourth participant in the study was a 71-year-old male (case 4) who was afflicted with PD for 4 years. After implantation of hOM-MSCs, there was a significant reduction in the oral maintenance dose of levodopa from 1.5 g/d before surgery to 0.25 g/d. The patient exhibited improvements in mood, activities of daily living, motor tests, and treatment complications (Additional file 6: Video S4). One month after treatment, the total UPDRS score decreased from 101 to 55. Additionally, the Hoehn and Yahr rating scale improved from level 4 to level 2 and the Schwab and England daily activity scale showed recovery from 30 to 60%. The results of 6-month follow-up post-surgery revealed a UPDRS total score of 81, a Hoehn and Yahr rating scale of level 3, and a Schwab and England daily activity scale of 50% (Additional file 2: Table S5). Case 4 exhibited good tolerance to the procedure with no severe adverse events reported during the post-procedure period.

Finally, the fifth participant in the cohort was a 62-year-old female (case 5) who was diagnosed with PD for 13 years. Following hOM-MSCs implantation, the oral maintenance dose of levodopa was significantly reduced from 1.5 g/d before surgery to 0.5 g/d. The patient showed improvements in mood, activities of daily living, and motor tests (Additional file 7: Video S5). One month after treatment, the total UPDRS score decreased from 75 to 53. Additionally, the Hoehn and Yahr rating scale improved from level 3 to level 2 and the Schwab and England daily activity scale exhibited recovery from 50 to 70%. The results of 6-month follow-up post-surgery revealed a UPDRS total score of 70, a Hoehn and Yahr rating scale of level 3, and a Schwab and England daily activity scale of 60% (Additional file 2: Table S6). Case 5 exhibited good tolerance to the procedure with no severe adverse events reported during the post-procedure period.

The Western blotting results revealed an upregulation in the protein expressions of TGF-β1 and CD206, while IL-1β was downregulated at 5 and 11 d post cell transplantation. These changes exhibited a time-dependent gradient (Fig. 8b). The ELISA results revealed a significant elevation in dopamine (DA) levels, as well as the presence of anti-inflammatory factors TGF-β1, IL-4, and IL-10 following cell therapy when compared to pre-transplantation levels at 5 and 11 d post-cell transplantation. Moreover, there was a notable reduction in the concentrations of proinflammatory cytokines IL-1β and TNF-α at 5 and 11 d post-cell transplantation. Both changes also exhibited a time-dependent gradient (Fig. 8c, d, Additional file 1: Fig. S7a, b). In general, the neurological function of 5 patients with PD was effectively improved following treatment with hOM-MSCs without any serious adverse events during the post-procedure period.

## Discussion

The pathologic mechanism underlying PD development needs to be further elucidated. The main pathologic marker of PD is α-Syn. Under normal physiologic conditions, there exists a dynamic equilibrium between the production and degradation of α-Syn. However, this balance is disrupted by various genetically or environmentally controlled pathologic factors, leading to misfolding of α-Syn [26]. In addition to the abnormal accumulation in the brain, α-Syn can also be transmitted to the brain via the vagus nerve from its abnormal accumulation starting in the gut [27]. During disease progression, α-Syn spreads to different regions of the brain in a prion-like manner [28]. Microglia are the first immune defense system of the human brain and one of the main cell types involved in the inflammatory response in the CNS [29]. Microglia can recognize misfolded α-Syn through Toll-like receptor (TLR) 2 and TLR4 on the cell surface, which subsequently initiates related signaling pathways. This process includes activation of the NF-κB signaling pathway and upregulation of proinflammatory cytokines expression, ultimately promoting a shift from an anti-inflammatory M2 phenotype to a proinflammatory M1 phenotype [30, 31]. Under pathologic conditions associated with PD, activation of the NF-κB signaling pathway in microglia promotes the secretion of inflammatory chemokines, such as RANTES and eotaxin. This leads to increased infiltration of CD8^+^ T cells into the SN region of the brain affected by PD and enhances cytotoxicity against dopaminergic neurons [32, 33]. Additionally, α-Syn can be internalized by microglia and acts as an antigen-presenting cell, which subsequently presents antigens to CD4^+^ and CD8^+^ T cells, triggering the activation and autoimmune response through multiple pathways that ultimately result in the apoptosis of dopaminergic neurons [11]. The regulation of microglia by T cells infiltrating the brain of PD patients is also an important aspect of pathogenesis. During the initial stage of the disease, the compromised blood–brain barrier permits the infiltration of T lymphocytes, including T regulatory cells (Tregs), into the brain. As the disease progresses, there is homeostatic dysregulation of Tregs and T effector cells (Teffs), leading to Teff-mediated activation via the secretion of diverse proinflammatory factors that upregulate MHC-II expression in microglia [34, 35]. Furthermore, heightened activation of microglia amplifies the impact on T cells, thereby engendering a deleterious feedback loop that expedites mitochondrial dysfunction and apoptosis in dopaminergic neurons [35]. Therefore, we propose the concept of a neuroimmune microenvironment in the pathogenesis and progression of PD. The key components that constitute the neuroimmune microenvironment of PD include persistent inflammatory response, activated microglia, an imbalance between Teffs and Tregs, as well as degeneration of dopaminergic neurons, which collectively contribute to the onset and progression of PD [36].

PD exerts psychological, physical, economic, and social effects on individuals with PD, as well as their families and society at large. Currently, there are no treatments available to cure PD or alter its progression of PD. As such, approaches for treating and preventing PD are urgently needed. Recently, the transplantation of MSCs has been effective in treating neurodegenerative diseases [37]. The degeneration of dopaminergic neurons is caused by the interaction of α-Syn, microglia, and T cells during PD [36]. As a result, if one or more of these links can be targeted to slow down disease progression, it will be a crucial strategy for PD prevention and treatment. In summary, the use of MSCs can accelerate the clearance and degradation of α-Syn via multitarget disease-modifying mechanisms, such as modulating microglia activation, enhancing the autophagy process, mediating endocytosis, protease secretion, and preventing pyroptosis of microglia [24, 37–40]. As cell-based therapy candidates for PD treatment, MSCs offer a broader scope beyond simple cell replacement due to their ability to detoxify ROS, as well as supply neurotrophic factors via paracrine effects that protect the mitochondrial function of dopaminergic neurons [24, 36]. Based on progress in understanding the MSC multitarget disease-modifying effect, we believe that MSCs could serve as an important therapeutic strategy or preventive measure against the onset of PD in high-risk individuals while also slowing down its progression.

Numerous studies have demonstrated the positive effects of stem cells, such as umbilical cord MSCs, bone marrow-derived MSCs, adipose-derived MSCs, or embryonic stem cells in PD treatment, which could improve neurologic function via diverse immunologic and histologic measures [41–44]. Nevertheless, these cells face several limitations that hinder their clinical application, including ethical issues, low yield rates, and immune rejection. To obtain more ideal nerve repair cells, our team successfully cultured OM-MSCs and established a comprehensive culture system. These cells exhibit a higher rate of proliferation and shorter passage time. Moreover, OM-MSCs are widely distributed in the nasal cavity with easy accessibility. They do not elicit immune rejection, nor pose significant ethical issues. Additionally, they retain biological activity that remains unchanged with age [23, 45]. As previously stated, the nasal mucosa originates from embryonic ectoderm and can directly differentiate into neurons under the right conditions. According to chromosome karyotype and tumor gene analyses, there is no genetic variation after extensive in vitro passages [46]. Our findings confirmed that OM-MSCs possess multi-lineage differentiation potential including neurons, bone tissue, and adipose tissue [23, 47]. Thus, the benefits of OM-MSCs make them an ideal source of cells for treating neuroimmune diseases like PD.

Selecting and confirming better experimental models was an important part of this study. The prefabricated fiber body-activated α-Syn, microglia cells (BV2), and neurons (SH-SY5Y) were used to construct the neurocellular immune model of PD, and simulate certain pathological processes of PD [24]. A chronic mouse model of PD was established by combining MPTP with probenecid [25]. Previous studies have shown that microglia cells in the SN of the MPTP chronic mouse model become activated, leading to a neuroinflammatory response [48, 49]. Therefore, MPTP combined with probenecid was selected in this study to construct a chronic PD model to mimic specific aspects of the neuroimmune model of PD. In this study, OM-MSCs were first amplified and cultured in vitro, and the 5th generation cells were chosen for identification, which showed the purity of the OM-MSCs exceeding 97%. After a 24-h co-culture with the PD cell model, it was observed that OM-MSCs have mitochondrial protective properties capable of promoting the transition from an M1-proinflammatory phenotype to an M2-antiinflammatory phenotype microglia cells while inhibiting the excessive autophagy reactions. These effects were significantly improved after hypoxic pretreatment. Subsequently, the OM-MSCs were transplanted into PD chronic mouse models via stereotactic lateral ventricles injection. Neural function scores indicated that hOM-MSCs substantially improved neurologic function in mice; moreover, hOM-MSC treatment resulted in less neurological damage when administered preventatively. Furthermore, hOM-MSCs significantly increased the immune and autophagy regulation of microglial cells in the PD model and protected the function of neuronal mitochondria.

The research focus of MSCs has shifted from cell replacement to multitarget therapy, such as paracrine and immune regulation. The generation of multifunctional factors by MSCs is a critical factor in their ability to modify the function of host cells. Studies have shown that the paracrine activity of cytokines secreted by MSCs effectively recovers and rescues the damage to neurologic function [24, 36]. However, it remains unclear which core functional factors secreted from hOM-MSCs play important roles in the hOM-MSC-modulated recovery of neurologic function. To further explore the underlying mechanism, the ATAC-seq technique combined with Iso-seq was employed to investigate the disparities in newly accessible chromatin regions and their corresponding transcripts, as well as the associated gene expression levels of OM-MSCs prior to and following hypoxia pretreatment. Bioinformatics analysis revealed significant upregulation in chromatin opening and transcription levels of LTBP1 in hOM-MSCs, along with a significant upregulation in TGF-β1 gene expression directly regulated by LTBP1. ISO-seq was performed on microglial cells derived from a PD cell model with the intervention from OM-MSCs. Based on the KEGG pathway classification, the significantly enriched results of DEGs and transcripts between the model and hOM-MSC intervention groups were analyzed, demonstrating that the PI3K-Akt signaling pathway was the most significantly enriched. Furthermore, immunomodulatory and neuroprotective mechanisms of hOM-MSCs were elucidated using a mouse model for PD treatment. scRNA-seq was employed to identify gene expression profiles in SN tissue following transplantation with hOM-MSCs for treatment and prevention purposes within the PD mouse model. Further analysis suggested that the PI3K-Akt/mTOR signaling pathway has a pivotal role in microglia during hOM-MSC treatment and prevention in a PD mouse model. Moreover, interactions between PTPRC-CD22, CD28-CD86, CD74-MIF, IL1B-ADRB2, and GPR37-PSAP receptors significantly impact the regulation of immunity and phagocytosis in microglial cells.

TGF-β1, a platelet-derived substance in the early 1980s, promotes the virus-like transformation and proliferation of tissue cells. Composing 391 amino acids, TGF-β1 is a precursor protein molecule that includes an active TGF-β1 molecule, signal peptide, and inactive associated peptide (LAP) [50]. Following secretion, TGF-β1 binds to LAP either covalently or non-covalently to form small precursor complexes devoid of activity. Subsequently, this complex is linked to LTBP1 via disulfide bonds to generate a large precursor complex that releases active TGF-β1 through integrin-mediated tension during cell contraction [51–53]. As a crucial cytokine for both innate and adaptive immunity, TGF-β1 plays roles in promoting neurogenesis, and gliosis, and regulating neural patterns, myelin and microglia development during brain maturation [51, 53]. It is worth noting that TGF-β1 is indispensable for microglia development and cell homeostasis. In the absence of TGF-β1, the *tmem119* and *p2ry12* genes responsible for maintaining homeostasis in microglia are downregulated, while the *Axl* and *APOE* genes associated with damage are upregulated [54]. Microglia cells serve as vital immune defense cells in the brain and play key roles in the neuroinflammatory response of the CNS, suggesting significant potential of TGF-β1 in PD and other neurodegenerative diseases. The TGF-β1 type I receptor, ALK, belongs to the RTK family, which mainly mediates cell signaling [55]. Upon activation, RTK forms dimers by binding to similar nearby kinases, and phosphorylation occurs at specific sites within the dimer. This phosphorylation event triggers the activation of ALK, enabling it to transfer phosphate groups to downstream proteins in the intracellular signaling pathway and ultimately leading to protein activation [55, 56].

The PI3K-Akt signaling pathway can be activated by various types of cytokine stimulation, thereby regulating fundamental cellular processes such as intracellular transcription, translation, proliferation, growth, and survival [57]. The binding of growth factors to their receptors (RTK or G-protein-coupled receptors) stimulates the activation of class Ia and Ib PI3K subtypes, respectively. Activation of PI3K produces phosphatidylinositol-3,4,5-triphosphate on the cell membrane, which in turn acts as a second messenger of downstream Akt activation [58]. The PI3K-Akt signaling pathway also plays an important role in the CNS, and its activation exhibits neuroprotective effects [59]. Studies have demonstrated that the MPTP-induced PD model significantly reduces the level of intracellular Akt phosphorylation, thus leading to apoptosis and loss of dopaminergic neurons [57, 59]. Furthermore, activation of the PI3K-Akt signaling pathway can downregulate the expression of inflammatory cytokines in microglia by regulating the NF-κB pathway [59].

The NF-κB protein is widely distributed throughout the nervous system and plays a pivotal role in regulating inflammatory response. It is highly expressed in striatal neurons of patients with PD [60]. The NF-κB protein family exists as homodimers or heterodimers, with p50/p65 being the most prevalent form [61]. In the progression of PD, damaged neurons release a large number of proinflammatory cytokines, such as IL-1β, IL-6, and TNF-α. These cytokines activate the NF-κB pathway in microglia cells, leading to neuroinflammation and extensive neuronal damage [60, 62]. Studies have shown that the inhibition of the NF-κB pathway alleviates the neuroinflammatory response and neuronal damage induced by MPTP in PD [60, 62]. Furthermore, activation of the NF-κB signaling pathway controls the transcription and expression of inflammatory chemokines like RANTES and eotaxin in various cell types. Studies have confirmed the activation of NF-κB in microglia cells of PD patients and the MPTP model in mice and monkeys, and have investigated the role of NF-κB in the adaptive immune response o in the nigra [63–65]. It can be concluded that under PD pathologic conditions, activation of the NF-κB signaling pathway in microglia promotes the secretion of inflammatory chemokines, such as RANTES and eotaxin, thus increasing the infiltration of CD8^+^ T cells into the SN of PD and enhancing the cytotoxic response to dopaminergic neurons [33]. This study confirmed that TGF-β1 secreted by hOM-MSCs downregulates the NF-κB pathway by activating the PI3K-Akt signaling pathway in microglia cells, thereby playing a role in immune regulation.

The mTOR is a crucial regulator of autophagy in mammals [66]. The PI3k-Akt pathway serves as the upstream regulatory pathway for mTOR, which gets activated by the receptors of neuro-nutrients and growth factors. This activation promotes cell growth, differentiation, and survival while suppressing cell apoptosis and other signals. Therefore, activation of the PI3K-Akt-mTOR pathway facilitates the protection of nerve cells and inhibits excessive autophagy through mTOR activation [67]. Moreover, this pathway plays an important regulatory role in the development of neurodegenerative diseases like PD and is essential for synaptic activity and normal function [68]. The interaction between the PI3K-Akt-mTOR pathway and the autophagy process, however, is very complex and presents a potential modulator function for autophagy. Therefore, the ideal protein factor or compound for improving PD should have the function of stimulating or maintaining the normal PI3K-Akt-mTOR survival pathway and regulating autophagy homeostasis of microglia cells. This study also confirmed that TGF-β1 secreted by hOM-MSCs downregulates autophagy-related proteins by activating PI3K-Akt-mTOR pathway signals, thus preserving the biological activity of microglia cells.

The present study investigated the impact of clinical-grade hOM-MSCs on neural functional recovery in both PD models and patients, as well as their potential for preventing PD in mouse models. We demonstrated that the core functional factor, TGF-β1, secreted from hOM-MSC plays critical roles in hOM-MSC-modulated recovery of mitochondrial function in dopaminergic neurons by improving microglia immune regulation and autophagy homeostasis in the SN, which are all closely associated with neuroinflammatory responses. Based on scRNA-seq, ATAC-seq combined with ISO-seq analysis, and a functional assay, we found that improvement in the neuroinflammatory response, as well as recovery of neural function upon exposure to hOM-MSCs, are at least partially mediated by TGF-β1 in the SN of PD through the activation of the ALK-PI3K-Akt signaling pathway in microglia. Additionally, the study has certain limitations regarding cell survival rate, number of homing cells, and immunomodulatory effects following MSC implantation in vivo; these factors play pivotal roles in determining the success of MSC therapy [36]. Excessive inflammatory response, oxidative stress, hypoxia, and other negative factors present at the site of injury may significantly impede MSC survival and efficacy [23]. Although we conducted hypoxic preconditioning on OM-MSCs to enhance their adaptability to hypoxia and other microenvironments, we did not further investigate the impact of PD nerve immune microenvironment in vivo on the survival rate, duration of survival, and homing capacity after transplantation. Therefore, the subsequent investigation will primarily focus on exploring the interactive regulatory effect and mechanism of MSCs within the neuroimmune microenvironment of PD. This research endeavor will also establish a solid theoretical foundation for developing standardized guidelines pertaining to MSC transplantation using PD animal models.

Furthermore, we have confirmed through a series of neurologic function scores and experiments that intraspinal transplantation of hOM-MSCs can improve the recovery of neurological function and regulate the neuroinflammatory response in PD patients without adverse reactions. It is noteworthy that all patients in the clinical trial exhibited significant improvement in neurological function within 7 d following cell transplantation. Based on the pre- and post-transplantation detection results of CSF and serum, we propose that this period is primarily attributed to the crucial immunomodulatory role played by hOM-MSCs’ paracrine effect. The majority of patients exhibited a certain degree of increase in neurological symptoms and scores at the 6-month follow-up, compared to 1 month after cell transplantation. These aforementioned reasons deserve careful consideration. MSCs have been widely utilized for the prevention and treatment of inflammatory and immune-related diseases owing to their robust immunomodulatory functions. However, the potential effectiveness of MSCs in cell therapy also relies on the activity and functional status. Stem cell senescence is a pivotal factor that significantly affects cellular activity and function, and diminishes the efficacy of cell therapy [69]. The local microenvironment for cell survival plays a crucial role in determining cellular activity and function. Several studies have confirmed that the inflammatory microenvironment is one of the key factors inducing stem cell senescence [70, 71]. PD patients exhibit sustained chronic neuroinflammation in the nigrostriatal region. Even if autologous MSCs are transplanted, they still need to withstand the impact of the inflammatory microenvironment upon entering the brain, which is likely to result in aging and even apoptosis due to continuous exposure to this microenvironment. We believe that the optimal approach to address this issue is currently through a combination of other drugs capable of regulating the inflammatory response or replenishing the number of MSCs within their effective period. We recommend that patients with PD undergo a second round of cell maintenance therapy within 6 – 12 months following their initial treatment. The safety and efficacy of intravenous cell transplantation are currently being investigated, particularly for the elderly population with PD, aiming to alleviate the burden of lumbar puncture. Additionally, strategies to enhance its effectiveness by increasing the number of cell transplantations are also under exploration. Furthermore, it is important to note that this clinical trial had certain limitations due to a small patient cohort and the absence of a randomized control group. Thus, the enrollment of participants in clinical trials involving hOM-MSCs for PD patients will be further expanded, and regular follow-up will be conducted with those who have previously undergone cell transplantation. Finally, although MSC therapy has demonstrated promising results in pre-clinical studies for various diseases, numerous challenges have emerged during clinical trials. Currently, there is a lack of international consensus on the clinical application of MSCs, including optimal cell dosage, administration intervals, culture conditions, and routes of administration. Therefore, further investigation into these factors as well as biological characteristics of MSCs derived from different sources is necessary to identify the most effective treatment strategies for diverse diseases and maximize potential benefits.

## Conclusions

To the best of our knowledge, this study represents the first attempt to investigate the effects of clinical-grade hOM-MSCs on neural functional recovery in both PD models and patients, as well as their potential preventive effects in mouse models of PD. Collectively, the data demonstrated that the core functional factor TGF-β1 secreted from hOM-MSC plays critical roles in hOM-MSC-modulated recovery of mitochondrial function in dopaminergic neurons by improving microglia immune regulation and autophagy homeostasis in the SN, which are all closely associated with neuroinflammatory responses. Mechanistically, exposure to hOM-MSCs enhances neuroinflammatory response and neural functional recovery partially through TGF-β1-mediated activation of the ALK-PI3K-Akt signaling pathway in microglia within the SN of PD patients. Furthermore, intraspinal transplantation of hOM-MSCs is confirmed to improve the recovery of neurologic function and regulate the neuroinflammatory response in PD patients. Hence, we suggest that treatment and prevention using hOM-MSCs could be a promising and effective neuroprotective candidate for PD and other progressive inflammatory-related neurodegenerative diseases. These findings raise the possibility that TGF-β1 may be used alone or combined with hOM-MSCs for PD treatment.

### Supplementary Information

**Additional file 1:** Materials and methods. **Fig. S1** Identification and biological characteristics of OM-MSCs. **Fig. S2** The hOM-MSCs enhanced mitochondrial function in neurons and the immunomodulation of microglia in the PD cell model, related to Fig. 1. **Fig. S3** Construction and evaluation criteria of a chronic mouse model of PD. **Fig. S4** The hOM-MSCs facilitated the recovery of nerve function and the immunomodulation of microglia in PD mouse model, related to Fig. 2. **Fig. S5** ScRNA-seq, ATAC-seq combined with ISO-seq to elucidate the underlying mechanisms of hOM-MSCs in PD models, related to Figs. 3 and 4. **Fig. S6** TGF-β1 mediates hOM-MSC to facilitate microglial immunomodulation by activating the PI3K-Akt signaling pathway, related to Figs. 5 and 6. **Fig. S7** Phase I hOM-MSC implantation clinical trial for PD patients, related to Fig. 7.

**Additional file 2:** **Table S1** The characteristics of the patients. **Table S2** Neurological function score in case 1. **Table S3** Neurological function score in case 2. **Table S4** Neurological function score in case 3. **Table S5** Neurological function score in case 4. **Table S6** Neurological function score in case 5.

**Additional file 3:** Video S1.

**Additional file 4:** Video S2.

**Additional file 5:** Video S3.

**Additional file 6:** Video S4.

**Additional file 7:** Video S5.

## Data Availability

In this study, the raw data are available from the corresponding author (Ming Lu) upon reasonable request and through collaborative investigations. All other data needed to evaluate the conclusions in the paper are presented in the paper or the supplementary materials.

## Abbreviations

AAV: Adeno-associated virus
ADRB2: Adrenoceptor beta 2
Akt: Protein kinase B
ALK: Anaplastic lymphoma Kinase
α-Syn: α-Synuclein
ATAC-seq: Assay for transposase-accessible chromatin with high-throughput sequencing
CD: Cluster of differentiation
CFT: 2β-Carbomethoxy-3β-(4-fluorophenyl)-(N-11C-methyl) tropane
CNS: Central nervous system
CSF: Cerebrospinal fluid
DA: Dopamine
DAPI: 4, 6-Diamino-2-phenyl indole
DAT: Dopamine transporter
DEGs: Differentially expressed genes
ECs: Endothelial cells
FITC: Fluorescein isothiocyanate isomer I
GFP: Green fluorescent protein
GPR37: G protein-coupled receptor 37
HVA: Homovanillic acid
hOM-MSC: Hypoxia-preconditioned olfactory mucosa mesenchymal stem cells
IGV: Integrative genomics viewer
IL: Interleukin
ISO-seq: Isoform-sequencing
JC-1: 5,5',6,6'-Tetrachloro-1,1',3,3'-tetraethylbenzimidazolcarbocyanine iodide
KEGG: Kyoto Encyclopedia of Genes and Genomes
LAMP-2: Lysosome-associated membrane protein-2
LTBP1: Latent transforming growth factor beta binding protein 1
LBs: Lewy bodies
MHC-II: Major histocompatibility complex class II
MIF: Migration inhibitory factor
MPs: Mononuclear phagocytes
MPTP: 1-Methyl-4-phenyl-1,2,3,6-tetrahydropyridine
MSCs: Mesenchymal stem cells
mTOR: Mammalian target of rapamycin
NeuN: Neuron-specific nuclear protein
NF-κB: Nuclear factor kappa-B
nOM-MSC: Normoxia-olfactory mucosa mesenchymal stem cells
OM-MSC: Olfactory mucosa mesenchymal stem cells
ONT: Oxford Nanopore Technologies
PBS: Phosphate buffer saline
PD: Parkinson's disease
PET: Positron Emission Tomography
PI: Propyridine iodide
PI3K: Phosphoinositide 3-kinase
PSAP: Prosaposin
PTPRC: Protein tyrosine phosphatase receptor type C
ROS: Reactive oxygen species
RTK: Receptor tyrosine kinase
scRNA-seq: Single-cell RNA sequencing
SN: Substantia nigra
STRO-1: Stromal cell antigen-1
SUV: Standard uptake value
TEM: Transmission electron microscopy
TGF-β1: Transforming growth factor-β1
TH: Tyrosine hydroxylase
TNF-α: Tumor necrosis factor-α
TSPO: Transporter protein
UPDRS: Unified Parkinson’s disease rating scale
UMAP: Uniform manifold approximation and projection
α-Syn: Alpha-synuclein
