# Supplementary material for: TGF-β1 mediates hypoxia-preconditioned olfactory mucosa mesenchymal stem cells improved neural functional recovery in Parkinson’s disease models and patients
Source: Mil Med Res. 2024 Jul 22;11:48. doi: 10.1186/s40779-024-00550-7 (PMC11265117; doi:10.1186/s40779-024-00550-7)
Supplement: Supplementary file 1 — Additional file 1: Materials and methods. Fig. S1 Identification and biological characteristics of OM-MSCs. Fig. S2 The hOM-MSCs enhanced mitochondrial function in neurons and the immunomodulation of microglia in the PD cell model, related to Fig. 1. Fig. S3 Construction and evaluation criteria of a chronic mouse model of PD. Fig. S4 The hOM-MSCs facilitated the recovery of nerve function and the immunomodulation of microglia in PD mouse model, related to Fig. 2. Fig. S5 ScRNA-seq, ATAC-seq combined with ISO-seq to elucidate the underlying mechanisms of hOM-MSCs in PD models, related to Figs. 3 and 4. Fig. S6 TGF-β1 mediates hOM-MSC to facilitate microglial immunomodulation by activating the PI3K-Akt signaling pathway, related to Figs. 5 and 6. Fig. S7 Phase I hOM-MSC implantation clinical trial for PD patients, related to Fig. 7. [file 40779_2024_550_MOESM1_ESM.pdf]

Materials and methods

Adipogenic differentiation

Inoculate 5×10^4 cells into the wells of a 6-well plate with added culture medium, and replace them with adipogenic differentiation medium (A1007001, Adipogenesis Differentiation Kit, Gibco, USA) the next day after adhesion. According to the manufacturer's instructions, replace the medium every 3 – 4 d and allow the cells to differentiate for 21 d. Remove the culture medium and wash with PBS 3 times. Fix the cells at room temperature for 10 min and wash the wells three additional times with PBS. Add Oil Red O solution to each well and incubate for 10 – 20 min. Wash multiple times with PBS to remove excess dye and observe under a microscope.

Osteogenic differentiation

Inoculate 5×10^4 cells into the wells of a 6-well plate with added culture medium, and replace them with complete osteogenic differentiation medium (A1007201, Osteogenic Differentiation Kit, Gibco, USA) the next day after adhesion. According to the manufacturer's instructions, replace the medium every 3 – 4 d and allow the cells to differentiate for 21 d. Remove the culture medium and wash with PBS 3 times. Fix the cells at room temperature for 10 min and wash the wells three additional times with PBS. Add 40 mmol/L of Alizarin Red solution to each well and incubate for 30 min. Wash multiple times with PBS to remove excess dye and observe under a microscope.

Fluorescence-activated cell sorting (FACS) analysis

OM-MSCs from the 4th passage were collected, washed 3 times, and then prepared for transplantation. Before on-board testing, OM-MSCs are incubated separately with flow cytometry antibodies CD34 (1:10; 12-0349-42, eBioscience, USA), CD44 (1:10; 12-0441-81, eBioscience, USA), CD45 (1:10; 12-0459-42, eBioscience, USA), CD73 (1:10; 12-0739-42, eBioscience, USA), CD90 (1:10; 12-0909-42, eBioscience, USA), CD105 (1:10; 12-1057-42, eBioscience, USA), CD133 (1:10; 12-1338-42, eBioscience, USA) and CD146 (1:10; 12-1469-41, eBioscience, USA) in the dark for 30 min. Standard FACS analysis was performed by flow cytometer (Beckman, USA) and analyzed by FlowJo software. Unstained or phenotype control samples were utilized to establish appropriate gates, voltages, and compensations for multivariate flow cytometry.

Apoptosis assay

Annexin V-FITC/propidium iodide (PI) apoptosis detection kit (KGA108, KeyGen, China) was used to monitor the apoptosis percentage in SH-SY5Y cells. The cells were incubated with 5 μl FITC-Annexin V and 5 μl PI in the dark at room temperature for 10 min. The rates of cellular apoptosis were recorded using a flow cytometer (Beckman, CA, USA) and analyzed by FlowJo software.

Mitochondrial membrane potential assay

The mitochondrial electrochemical gradient was detected in SH-SY5Y cells using MitoProbe (the cationic dye JC1, 5′,6,6′-tetrachloro-1,1′,3,3-tetraethyl benzimidazole-carbocyanine iodide, Beyotime Biotech. Inc, China). Briefly, the SH-SY5Y cells in each group were collected and incubated with prewarmed 5,5',6,6'-tetrachloro-1,1',3,3'-tetraethylbenzimidazolcarbocyanine iodide (JC-1) for 30 min at 37 °C in the dark. After three washes with prewarmed phosphate buffer saline (PBS), the fluorescence intensity of the cells was measured using a flow cytometer (Beckman, USA) and analyzed by FlowJo software.

Cell processing and immunofluorescence

Cellular immunofluorescence staining was fixed with 4% paraformaldehyde (PFA) for 20 min. After washing with PBS, the cells were blocked with a blocking solution consisting of 1% Triton X-100 and 4% bovine serum albumin (BSA) in PBS at 4 °C for 20 min and then incubated with the first antibody diluted in a closed solution at 4 °C for overnight. The next day, they were stained with the corresponding secondary antibody for 1 h. Stained cells were counterstained with 4’,6-diamidino-2-phenylindole (DAPI; 1:1000; D9542, Sigma, USA) and mounted with an anti-fluorescence quenching sealing solution (P0126, Beyotime, China). Finally, slides were observed under a fluorescence microscope (Leica, Germany).

Histological analysis

One week after cell transplantation, animals were perfused transcardially with PBS followed by PFA. The whole brain of the mouse was dissected and fixed in 4% (v/w) formaldehyde for 48 h. Subsequently, the brain was embedded in paraffin and cut into thick slices of 5 μm.

Standard staining techniques were used with primary antibodies against Iba1 (1:100; ab178847, Abcam, USA). The slices were placed flat and incubated overnight at 4 °C in a humid chamber. Afterward, the slides were placed in PBS (pH 7.4) and washed 3 times on a decolorizing shaker for 5

min each time. Following that, the corresponding Cy3 conjugated Goat Anti-Rabbit IgG (1:200; GB21303, Servicebio, China) secondary antibody was added and incubated at room temperature for 50 min in the dark. After washing 3 times, the DAPI staining solution was applied and allowed to incubate at room temperature for 10 min in the dark. Finally, the glass slides were sealed and observed under a fluorescence microscope (Leica, Germany).

For tyrosine hydroxylase (TH) immunohistochemistry analysis, the sections were washed with PBS before antigen retrieval by pouring over them hydrochloric acid to a concentration of 1 mol/L over minutes. Then, to penetrate the cell membrane, 0.3% Triton was used. Samples underwent another round of washing with PBS followed by blocking the secondary antibody response using 10% goat serum for 30 min. The diluted primary antibody (1:200; ab137869, Abcam, USA) was then added to the sample and placed at a temperature of 2 – 8 °C for one night. The next day, the secondary antibody [FITC Anti-Mouse IgG2a heavy chain (1:200; ab97244, Abcam, USA)] was added to samples and incubated at 37 °C for 1.5 h in the darkness. Subsequently, DAPI staining was used. Finally, the samples were examined by the Olympus fluorescent microscope (400× magnification).

Western blotting

The collected cells or SN of C57BL/6 mice were processed for Western blotting analysis as described previously [1]. Proteins were transferred onto a PVDF membrane (Millipore, USA) once the bromophenol blue reached the bottom. The blots were blocked in a 4% BSA solution in TBST (0.05%) for 1 h at room temperature and then incubated overnight at 4 °C with the corresponding primary antibody. After incubation with secondary antibodies at room temperature for at least 1 h, the blot was visualized by the ChemiDoc XRS imaging system.

ELISA

The levels of interleukin (IL)-1β, tumor necrosis factor (TNF)-α, IL-4, IL-10, dopamine (DA), and homovanillic acid (HVA) in the cell culture supernatants of OM-MSCs and SN tissues from C57BL/6 mice were quantified using an ELISA kit following the manufacturer’s instructions. After incubation with a working solution, samples were subjected to reaction with substrate solution at 37 °C for 0.5 h. The absorbance was measured at 450 nm using a microplate reader (Thermo Fisher, USA) after the addition of the termination solution.

Scanning electron microscopy (SEM) and transmission electron microscopy (TEM)

Dehydrate the cells in a graded series of ethanol and dry with hexamethyldisilane. After gold plating (Quorum Q150 RES; USA), observe the cell morphology using an SEM (300VP; Carl Zeiss, Germany). For TEM analysis, the collected cells or SN of C57BL/6 mice were fixed overnight at 4 °C with 2.5% glutaraldehyde, followed by washing in cacodylate buffer and fixation with 1% osmium tetroxide. After another round of washing in cacodylate buffer, the samples were embedded in a double-staining solution containing 5% uranyl acetate and lead citrate before being sectioned using an ultramicrotome for examination under an electron microscope (HT7700, Hitachi, Tokyo, Japan).

Behavioral test

The mice should be allowed to adapt to the laboratory environment for 2 h in advance and then placed in the center of the laboratory (50 cm × 50 cm × 25 cm). During the test, an automated tracking system will record the mice's trajectory, movement distance, speed, and time over a period of 5 min. To eliminate any potential interference from animal urine odor, it is necessary to clean the apparatus with 75% ethanol before and after each test.

We employed the rotarod test as an additional assessment of motor ability, such as motor coordination, balance, and motor learning, using an automated rotarod device (Shanghai Jiliang Software Science & Technology Co., Ltd., China). Before testing, the mice underwent daily training sessions lasting for 5 min daily at a constant speed of 10 rpm/min for 3 d. During the formal experiment, each mouse was placed on the accelerating rotating rod device. The speed was gradually increased from 4 rpm/min to 30 rpm/min within 3 min and then kept at 30 rpm/min for 5 min. Throughout the test duration, the instrument automatically records the distance and time. Exercise test data were calculated based on the average value of 3 tests.

Positron emission tomography-computed tomography (PET-CT)

Before undergoing PET imaging, mice were anesthetized with 2% isoflurane, and approximately (200 ± 10) μCi 2β-carbomethoxy-3β-(4-fluorophenyl)-(N-11C-methyl) tropane (CFT) or (50 ± 10) μCi [^{18}F]F-DPA was injected intravenously. After a period of 40 min following CFT ingestion, the mice were again anesthetized with 2% isoflurane and positioned on the scanning table. PET/CT images were acquired in static mode for 10 min, followed by normal mode CT scans using the TransPET Discoverist

180 system. The PET images were reconstructed utilizing the three-dimensional Ordered Subset Expectation Maximization method with a pixel size of 0.5 mm × 0.5 mm × 0.5 mm. Using the FDK algorithm, the CT image was reconstructed with a matrix size of 256 × 256 × 256 pixels. Images were displayed using the software AMIDE Medical Imaging Data Examiner and Pmod.

Use the software to analyze the PET image, outline the region of interest (ROI), and calculate the standard uptake value (SUV) value of each ROI. Calculation formula for SUV:

$$\mathrm{SUV} = \frac{C_{\mathrm{T}}}{D_{\mathrm{Inj}}} \times \frac{V_{\mathrm{T}}}{W_{\mathrm{T}}} \times W_{\mathrm{S}}$$

C_{T} represents the activity per unit volume of tissue, D_{Inj} represents the injection dose, V_{T} represents the volume of the tissue, and W_{T} represents the mass of the tissue, where $V_{\mathrm{T}}/W_{\mathrm{T}}$ represents the tissue density, and W_{S} represents the mouse's mass.

Assay for transposase-accessible chromatin with high-throughput sequencing (ATAC-seq)

Preparation of samples

Both normoxic and hypoxic groups of OM-MSCs were centrifuged with 50,000 cells for 5 min at 4 °C with a centrifugation force of 500 *g*. Subsequently, the supernatant was discarded and the cells were washed once with pre-cooled PBS using the same centrifugation parameters. After centrifugation, the supernatant was removed and another round of centrifugation was performed at 500 *g*. The cell suspension was then subjected to cold lysis buffer followed by centrifugation at 4 °C for 10 min at 500 *g* to remove the supernatant. The transposase reaction system was prepared using TN5 transposase, And the nuclei were suspended in this reaction system. After 30 min of incubation at 37 °C, DNA purification was carried out. Purified DNA served as input for PCR amplification in a configured PCR reaction system. Finally, after DNA purification on the Illumina platform, the resulting DNA library underwent correction.

Analysis

The original Reads obtained through ATAC-seq require de-adaptation and low-quality filtering prior to generating CleanReads. Subsequently, positional information for comparison is obtained through alignment with the reference genome sequence; identification of peak positions (Peaks) is achieved by

analyzing the genomic position information of Reads. Gene annotation is then performed on these peak positions to generate a list of genes affected by the peaks. Additionally, motif analysis can be performed on the identified Peak to identify potential transcription factor binding site information. In the case of multiple samples, differential peak comparisons are made to discover genes related to differential peaks. Functional enrichment analysis is subsequently performed on these genes in order to determine our target genes.

Isoform-sequencing (ISO-seq)

The cDNA library was prepared from 1 μg of total RNA using the cDNApcr Sequencing Kit (SQK-PCS109) provided by Oxford Nanopore Technologies (ONT, Britain). Briefly, reverse transcriptase with template-switching activity was used to enrich for full-length cDNA and add defined PCR adapters directly to both ends of the first-strand cDNA. The amplification of 14 loops was performed using LongAmp Tag (NEB). Subsequently, the PCR products were ligated to ONT adapters with T4 DNA ligase (NEB). Following the ONT protocol, DNA purification was performed using magnetic beads. Finally, the resulting cDNA library was added to the FLO-MIN109 cell flow and run on the PromethION platform from Biomarker Technologies (Beijing, China).

Single-cell RNA sequencing (scRNA-seq)

To elucidate the immunomodulatory and neuroprotective mechanisms of hOM-MSCs in a mouse model of PD, scRNA-seq was used to identify gene expression profiles in SN tissue obtained from the transplantation of hOM-MSCs in the PD mouse model. The PD model consisted of three groups: model 1, model 2, and model 3. The nor_MSC1, nor_MSC2, and nor_MSC3 groups received nOM-MSC treatment. The hyp_MSC1, hyp_MSC2, and hyp_MSC3 groups were administered hOM-MSC treatment, while the pre_MSC1, pre_MSC2, and pre_MSC3 were given hOM-MSCs for prevention purposes.

Tissue dissociation and preparation

The fresh tissues were stored in the sCelLive™ Tissue Preservation Solution (Singleron, China) on ice within 30 min after surgery. The specimens were washed 3 times with Hanks balanced salt solution, minced into small pieces, and then digested with 3 ml of sCelLive™ Tissue Dissociation Solution (Singleron, China) through the Singleron PythoN™ Tissue Dissociation System at 37 °C for 15 min.

The resulting cell suspension was collected and filtered through a 40-micron sterile strainer. Afterward, the GEXSCOPE® red blood cell lysis buffer (RCLB, Singleton, China) was added to the mixture [cell:RCLB = 1:2 (volume ratio)], which was then incubated at room temperature for approximately 5 – 8 min to remove red blood cells. The mixture was then centrifuged at 300 *g* at a temperature of 4 °C for 5 min to remove supernatant and gently suspended in PBS. Finally, Trypan Blue staining was applied to evaluate cell viability under microscopic examination.

Reverse transcription (RT), amplification, and library construction

Single-cell suspensions (2×10^5 cells/ml) with PBS (HyClone, China) were loaded onto a microwell chip using the Singleron Matrix® Single Cell Processing System. Barcoding beads are subsequently collected from the microwell chip. The captured mRNA on the barcoding beads was then reverse transcribed to obtain cDNA, which underwent PCR amplification. The amplified cDNA was fragmented and ligated with sequencing adapters. The scRNA-seq libraries were constructed according to the protocol of the GEXSCOPE® Single Cell RNA Library Kits (Singleton, China). Individual libraries were diluted to 4 nmol/L, pooled together, and sequenced on an Illumina novaseq 6000 platform with 150 bp paired-end reads.

Quality control, dimension-reduction, and clustering

Cells were filtered based on gene counts below 200 and the top 2% of both gene counts and unique molecular identifier counts. Cells with over 20% mitochondrial content were excluded. We used functions from Seurat v3.1.2 for dimension-reduction and clustering analysis, utilizing the top 20 principal components to separate cells into multiple clusters with the FindClusters algorithm. Batch effects between samples were removed by Harmony. Finally, a uniform manifold approximation and projection algorithm was applied to visualize cells in a two-dimensional space.

Differentially expressed genes (DEGs) analysis

To identify DEGs, the Seurat FindMarkers function was employed using the Wilcox likelihood-ratio test with default parameters. DEGs were selected based on their expression in more than 10% of the cells in a cluster and an average log (fold change) > 0.25.

Cell type annotation and pathway enrichment analysis

The cell type identity of each cluster was determined with the expression of canonical markers found in the DEGs using the SynEcoSys database. Heatmaps, dot plots, and violin plots were generated using Seurat v3.1.2 to visualize the expression patterns of these markers for identifying each cell type. To investigate the potential functions of DEGs, the Kyoto Encyclopedia of Genes and Genomes (KEGG) analysis was used with the "clusterProfiler" R package (v3.16.1). Pathways with a P_{adjust} value < 0.05 were considered significantly enriched.

Cell-cell interaction analysis

The analysis of cell-cell interaction was performed by CellPhoneDB v2.1.0 based on known receptor-ligand interactions between two specific cell types or subtypes. Individual expression levels of ligands or receptors were adjusted using a threshold value derived from the average log gene expression distribution across all the cell types. The significant cell-cell interactions were defined as *P*-value < 0.05 and average log expression > 0.1, which were visualized with the circle R package (v0.4.10).

RT-qPCR

Isolation of total RNA from cells was performed using the TRIzol reagent (Thermo Fisher Scientific, USA). In accordance with the manufacturer's instructions, RT-qPCR was performed using the UltraSYBR Mixture (Beijing ComWin Biotech Co., Ltd., China). The specific fragments were generated using the following qPCR primer sequences: 5′-ACCCTGAAGTACCCCATCGAG-3′ and 5′-AGCACAGCCTGGATAGCAAC-3′ for human β-actin, 5′-TAACCACACTGGCCGCATC-3′ and 5′-GTGGTGGTGTTCCCCTTCTC-3′ for human latent transforming growth factor beta binding protein 1 (LTBP1), and 5′-AGCAACAATTCCTGGCGATACCTC-3′ and 5′-CAATTTCCCCTCCACGGCTCA-3′ for human transforming growth factor-β1 (TGF-β1). The mRNA expression level of each target gene was normalized to β-actin expression, and relative expression was calculated according to the standard $2^{-\Delta\Delta Ct}$ method.

Cell shRNA transfection

The sequences encoding three different shRNAs targeting human TGFB1 and shTGFB1 (seq1, targeting: CAGCAACAATTCCTGGCGATA; seq2, targeting: AAGCAGAGTACACACAGCATA; seq3, targeting: GAGCCCTGGACACCAACTATT) were individually inserted into GV493 lentiviral construct provided by Genechem. Each of the constructs was co-transfected to HEK-293 cells (SCSP-

5209, National Collection of Authenticated Cell Cultures, China) along with lentivirus envelope constructs also obtained from Genechem. The generated lentiviral particles were then added to OM-MSCs.

Adeno-associated virus (AAV) construction and stereotaxic injection

Anaplastic lymphoma kinase (ALK) AAV was constructed by Genechem Company (Shanghai, China). AAV ALK targets the mouse microglia surface receptor ALK. Intracerebroventricular injection of AAV was carried out using a stereotaxic instrument. After mouse anesthesia, 3 μl AAV (1×10^{12} carrier genomes/ml) was injected with a microinjection pump at a constant rate of 1.0 μl/min. PD modeling was performed 1 week later.

References

1. Zhuo Y, Chen W, Li W, Huang Y, Duan D, Ge L, et al. Ischemic-hypoxic preconditioning enhances the mitochondrial function recovery of transplanted olfactory mucosa mesenchymal stem cells via miR-181a signaling in ischemic stroke. Aging. 2021;13:11234-56.

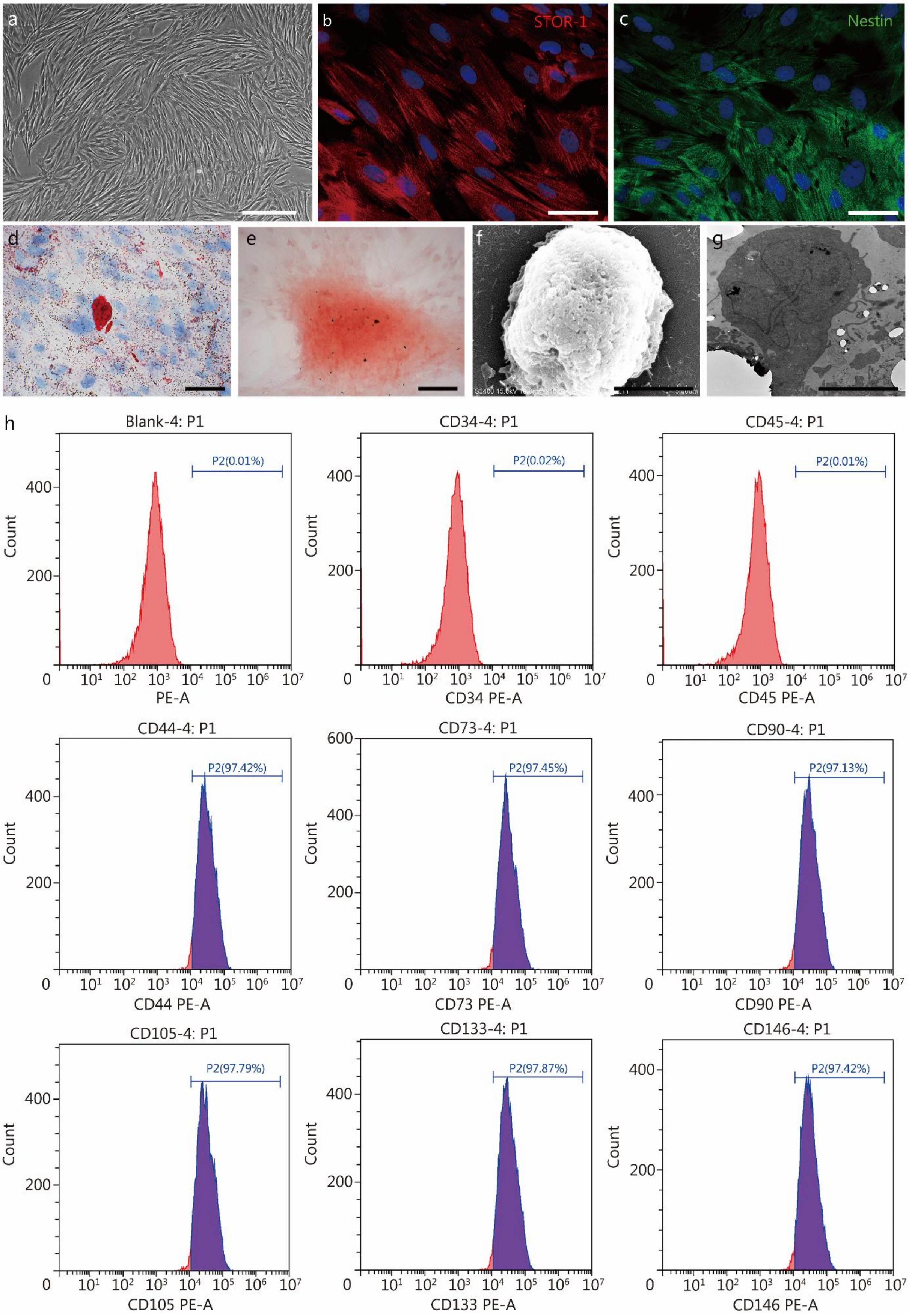

Fig. S1 Identification and biological characteristics of OM-MSCs**. a** The OM-MSCs exhibit a spindle-shaped morphology with a radial arrangement under light microscopy (scale bar = 100 μm). Exemplary

immunofluorescence micrograph showing the intracellular localization of STRO-1 (**b**) and Nestin (**c**) (scale bars = 40 μm). **d** Oil Red O staining showing red lipid droplets in the cytoplasm. **e** Alizarin Red staining showing red mineralized nodules in the cells. **f** Scanning electron microscope showing many microvilli on the surface of the OM-MSCs (scale bar = 5 μm) **g** TEM showing OM-MSCs were in a relatively active phase (scale bar = 5 μm). **h** Flow cytometry assays showing OM-MSCs had high expression of CD44, CD73, CD90, CD105, CD133, and CD146. OM-MSCs did not express CD34 and CD45, with an OM-MSC purity > 97%. OM-MSCs olfactory mucosa mesenchymal stem cells, CD recombinant cluster of differentiation, STRO-1 stromal cell antigen 1, TEM transmission electron microscopy

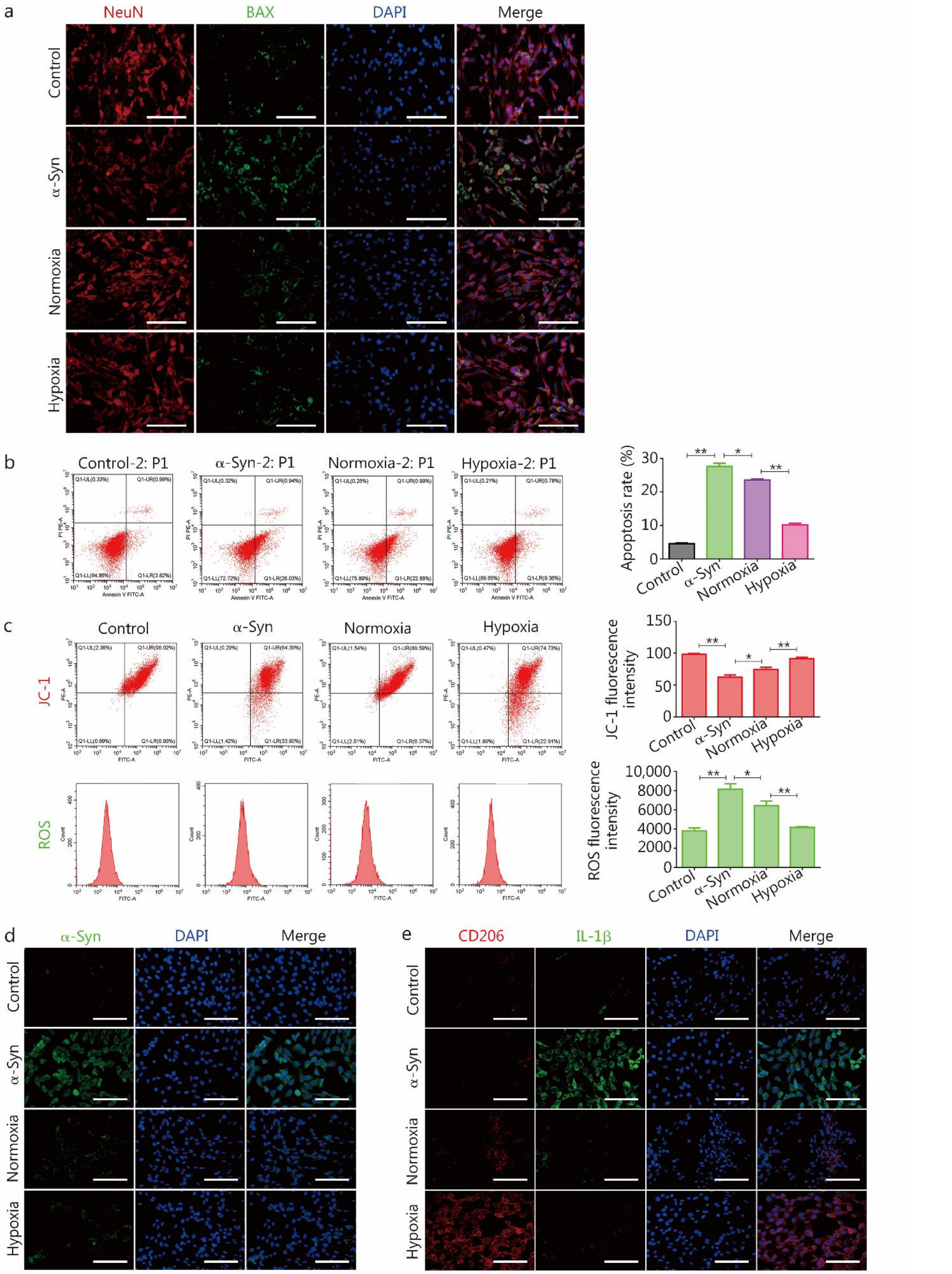

Fig. S2 The hOM-MSCs enhanced mitochondrial function in neurons and the immunomodulation of microglia in the PD cell model, related to Fig. 1. **a** Exemplary immunofluorescence micrograph showing NeuN, BAX, and nuclei (DAPI) expression in SH-SY5Y cells (scale bars = 40 μm). **b** Flow

cytometry assays showing apoptosis ratio in SH-SY5Y cells. **c** Flow cytometry assays to assess JC-1 and ROS, which are indicative of mitochondrial function. **d** Exemplary immunofluorescence micrograph showing α-synuclein (α-Syn) and nuclei (DAPI) expression in BV2 cells (scale bars = 40 μm). **e** Exemplary immunofluorescence micrograph showing CD206, IL-1β, and nuclei (DAPI) expression in BV2 cells (scale bars = 40 μm). Data are represented as mean ± SEM. $^{*}P < 0.05$, $^{**}P < 0.01$. NeuN neuron-specific nuclear protein, DAPI 4',6-diamidino-2-phenylindole, ROS reactive oxygen species, JC-1 5,5',6,6'-tetrachloro-1,1',3,3'-tetraethylbenzimidazolcarbocyanine iodide, IL-1β interleukin-1β, hOM-MSCs hypoxia-olfactory mucosa mesenchymal stem cells, CD recombinant cluster of differentiation

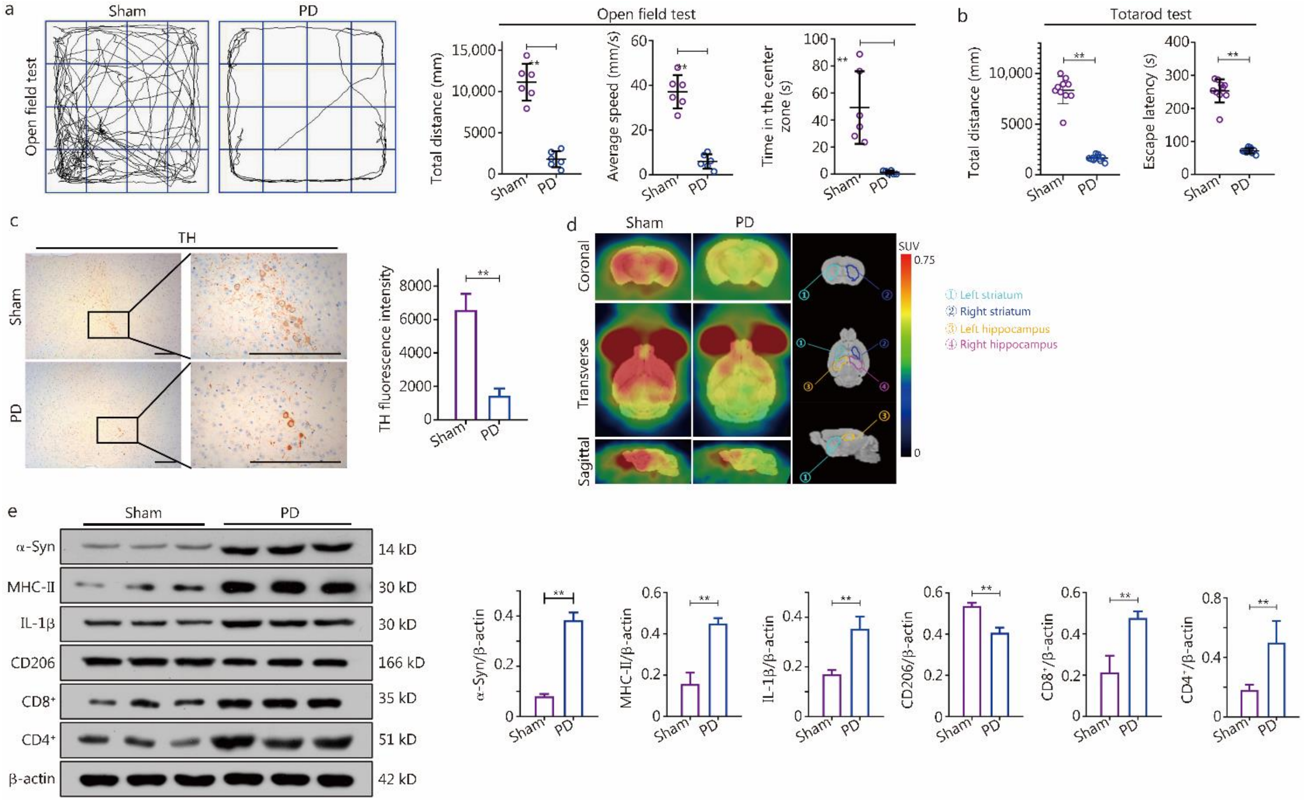

Fig. S3 Construction and evaluation criteria of a chronic mouse model of PD. **a** The neurologic function score of open field test showing the activity trace of PD mice, and histogram of total distance, average velocity, and central time (n = 6). **b** The neurologic function score of the Tatarod test shows the histogram of total distance and escape latency (n = 6). **c** Exemplary immunohistochemistry micrograph showing TH^+ expression in the SN of PD mice (scale bars = 200 μm). **d** CFT-PET brain imaging showing the distribution of DAT in brain tissue of PD mice. **e** Immunoblot measuringα-Syn, IL-1β, CD206, MHC-II, $CD8^+$, and $CD4^+$ protein expression in the SN of PD mice. Data are represented as mean ± SEM. $^{**}P < 0.01$. TH tyrosine hydroxylase, α-Syn α-synuclein, IL-1β interleukin-1β, PD Parkinson's disease, SN substantia nigra, CD recombinant cluster of differentiation, MHC-II major histocompatibility complex class II, CFT 2β-carbomethoxy-3β-(4-fluorophenyl)-(N-11C-methyl) tropan, PET positron emission tomography, SUV standard uptake value

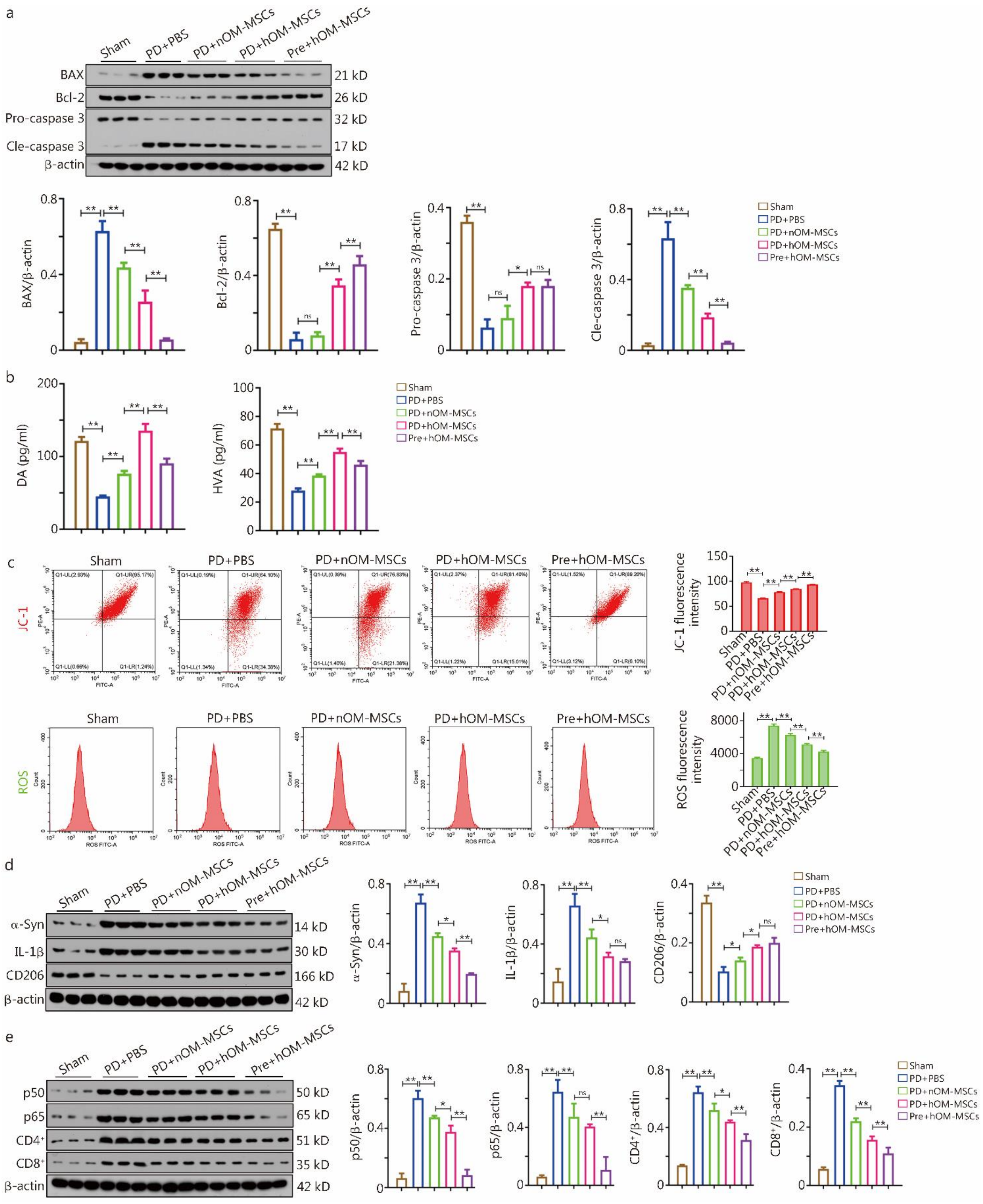

Fig. S4 The hOM-MSCs facilitated the recovery of nerve function and the immunomodulation of microglia in the PD mouse model, related to Fig. 2. **a** Western blotting measuring BAX, Bcl-2, and caspase 3 protein expression in the SN of PD mice. **b** ELISA showing a histogram of DA and HVA protein concentration in the SN of PD mice. **c** Flow cytometry assays to assess JC-1 and ROS, which are indicative of mitochondrial function. **d** Western blotting measuring α-Syn, IL-1β, and CD206

protein expression in the SN of PD mice. **e** Western blotting measuring p50, p65, $CD4^+$, and $CD8^+$ protein expression in the SN of PD mice. Data are represented as mean ± SEM. $^*P < 0.05$, $^{**}P < 0.01$, ns non-significant. hOM-MSCs hypoxia-olfactory mucosa mesenchymal stem cells, PD Parkinson's disease, SN substantia nigra, α-Syn α-synuclein, CD recombinant cluster of differentiation, HVA homovanillic acid, DA dopamine, IL-1β interleukin-1β, ROS reactive oxygen species, JC-1 5,5',6,6'-tetrachloro-1,1',3,3'-tetraethylbenzimidazolcarbocyanine iodide, PBS phosphate buffer saline, mono mononuclear

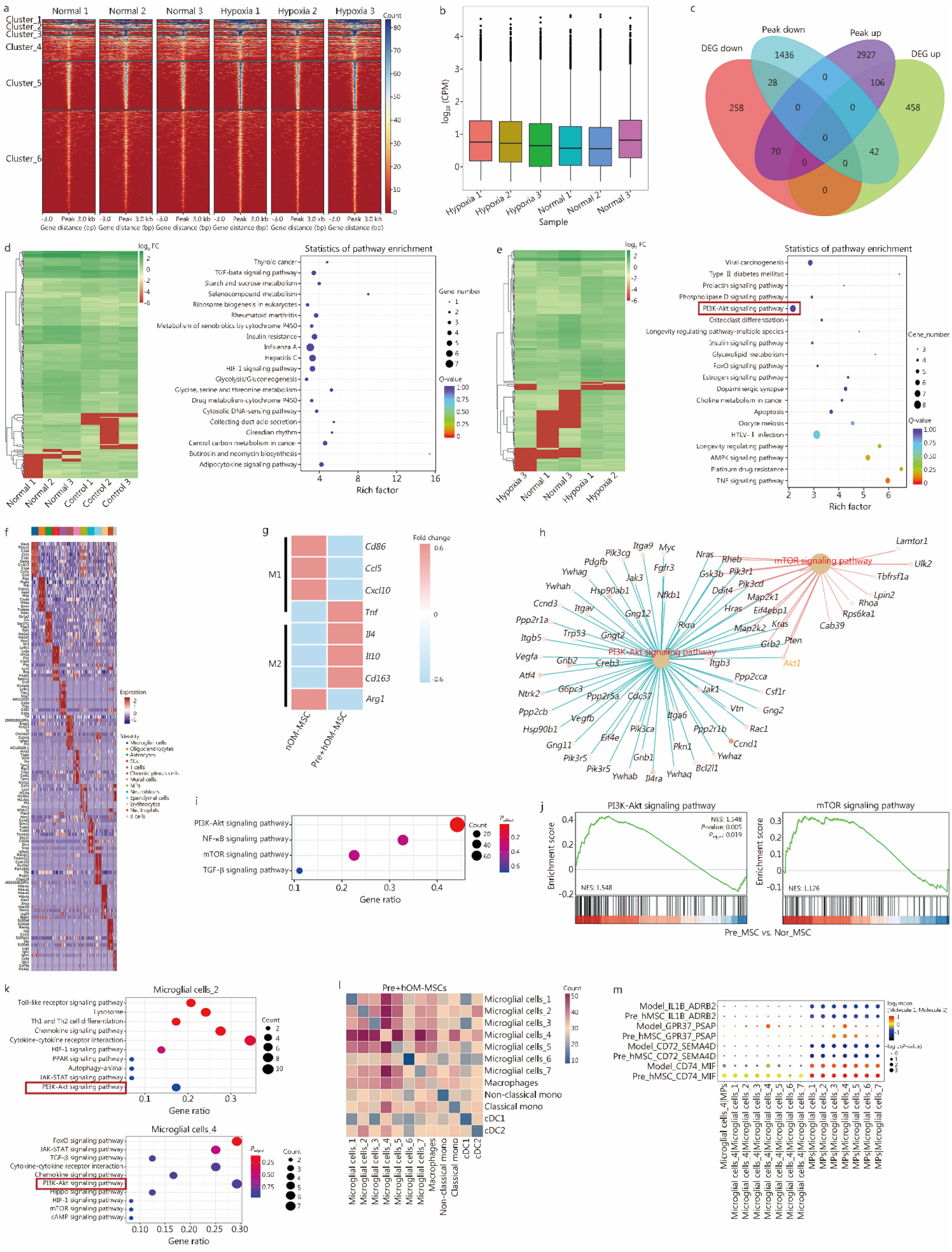

Fig. S5 ATAC-seq, scRNA-seq combined with ISO-seq to elucidate the underlying mechanisms of hOM-MSCs in PD models, related to Figs. 3 and 4. **a** DeepTools generates an ATAC-seq read enrichment heatmap for differential peaks in each sample group, facilitating intuitive grouping and

observation of differences between groups. **b** Boxplot showing the distribution of CPM to assess the discreteness of gene expression levels within a single sample. **c** Utilizing a Venn diagram for the analysis and visualization of co-occurring genes or specific changes in expression levels. **d** Differential gene enrichment heatmap before and after intervention with nOM-MSCs (left). Annotation results of DEGs were classified according to KEGG pathway types in the microglia of the PD cell model (right). **e** Differential gene enrichment heatmap after intervention with nOM-MSCs and hOM-MSCs (left). Annotation results of DEGs were classified according to KEGG pathway types in the microglia of the PD cell model (right). **f** Heatmap showing the genes expressed in > 10% of the cells in a cluster and with an FC value > 0.25 as DEGs. **g** Heatmap showing microglial M1 markers and M2 markers between nOM-MSCs treatment and hOM-MSCs prevention in the SN of PD mice. **h** GSEA showing the two significantly enriched pathways and the corresponding genes in the enrichment bubble map. **i** Gene network analysis revealed significant enrichment and interaction of PI3K-Akt/mTOR signaling pathway in microglia. **j** Line plots of enrichment score showed upregulation of PI3K-Akt/mTOR signaling pathway in microglia. **k** KEGG pathway dotplot showing the enrichment and up-regulation signaling pathway in microglial cells_2 and _4 in the SN of PD mice after hOM-MSCs prevention. **l** Heatmap showing the cell-cell interaction analysis based on known receptor-ligand interactions between all subtypes of microglia and MPs in the SN of PD mice after hOM-MSCs prevention. **m** Dot graph showing the cell-cell interaction differences between the four pairs of receptor-ligands were the most significant. hOM-MSCs hypoxia-olfactory mucosa mesenchymal stem cells, PD Parkinson's disease, nOM-MSCs normoxia-olfactory mucosa mesenchymal stem cells, DEGs differentially expressed genes, KEGG Kyoto Encyclopedia of Genes and Genomes, SN substantia nigra, MPs mononuclear phagocytes, ATAC-seq assay for transposase-accessible chromatin with high-throughput sequencing, ISO-seq isoform-sequencing, scRNA-seq single-cell RNA sequencing, CPM counts per million, FC fold change, GSEA Gene Set Enrichment Analysis, NES normalized enrichment score, ADRB2 adrenoceptor beta 2, PSAP prosaposin, SEMA4D recombinant semaphorin 4D, MIF migration inhibitory factor, FoxO forkhead box O, HIF-1 hypoxia inducible factor-1, JAK janus tyrosine kinase, STAT signal transducer and activator of transcription

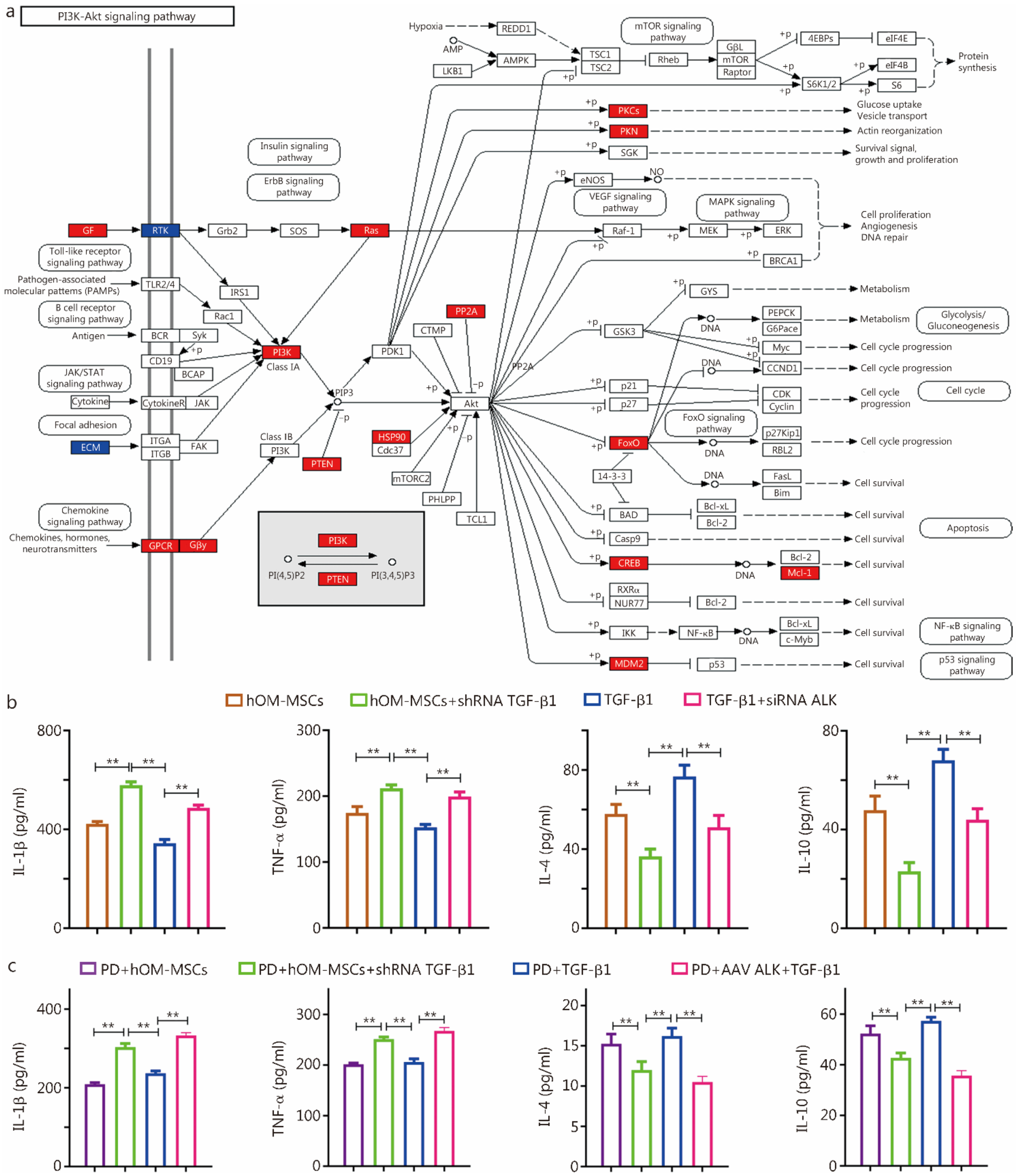

Fig. S6 TGF-β1 mediates hOM-MSCs to facilitate microglial immunomodulation by activating the PI3K-Akt signaling pathway, related to Figs. 5 and 6. **a** KEGG pathway map showing the extracellular growth factor in microglia after hOM-MSC intervention activates the PI3K-Akt signaling pathway through binding to the RTK receptor of the cell membrane. **b** ELISA showing histogram of IL-1β, TNF-α, IL-4, and IL-10 protein concentration in the supernatant of PD cell model. **c** ELISA showing histogram of IL-1β, TNF-α, IL-4, and IL-10 protein concentration in the SN of PD mice model. Data

are represented as mean ± SEM. $^{**}P < 0.01$. GF growth factor, RTK receptor tyrosine kinase, Grb2 growth factor receptor-bound protein 2, ErbB receptor tyrosine kinases, Ras rat sarcoma, SOS son of sevenless, ECM extracellular matrix, GPCR G protein-coupled receptor, PTEN phosphate and tension homology deleted on chromosome ten, HSP90 heat shock protein 90, PP2A protein phosphatase 2A, FOXO forkhead box O, PKN phosphorylation of protein kinase N, PDK1 phosphoinositide-dependent protein kinase-1, PKC protein kinase C, CREB cAMP response element binding protein, MDM2 p53 binding protein homolog, IKKs The IκB kinase, NF-κB nuclear factor kappa-B, Mcl-1 myeloid cell leukemia-1, ERK extracellular regulated protein kinases, JAK Janus kinase, FAK focal adhesion kinase, TLR Toll like receptor 2, Rac1 ras-related C3 botulinum toxin substrate 1, hOM-MSCs hypoxia-olfactory mucosa mesenchymal stem cells, KEGG Kyoto Encyclopedia of Genes and Genomes, TGF-β1 transforming growth factor-β1, IL-1β interleukin-1β, ALK anaplasticlymphoma kinase, PI3K phosphoinositide 3-kinase, Akt protein kinase B, TNF-α tumor necrosis factor-α, IL-4 interleukin-4, IL-10 interleukin-10

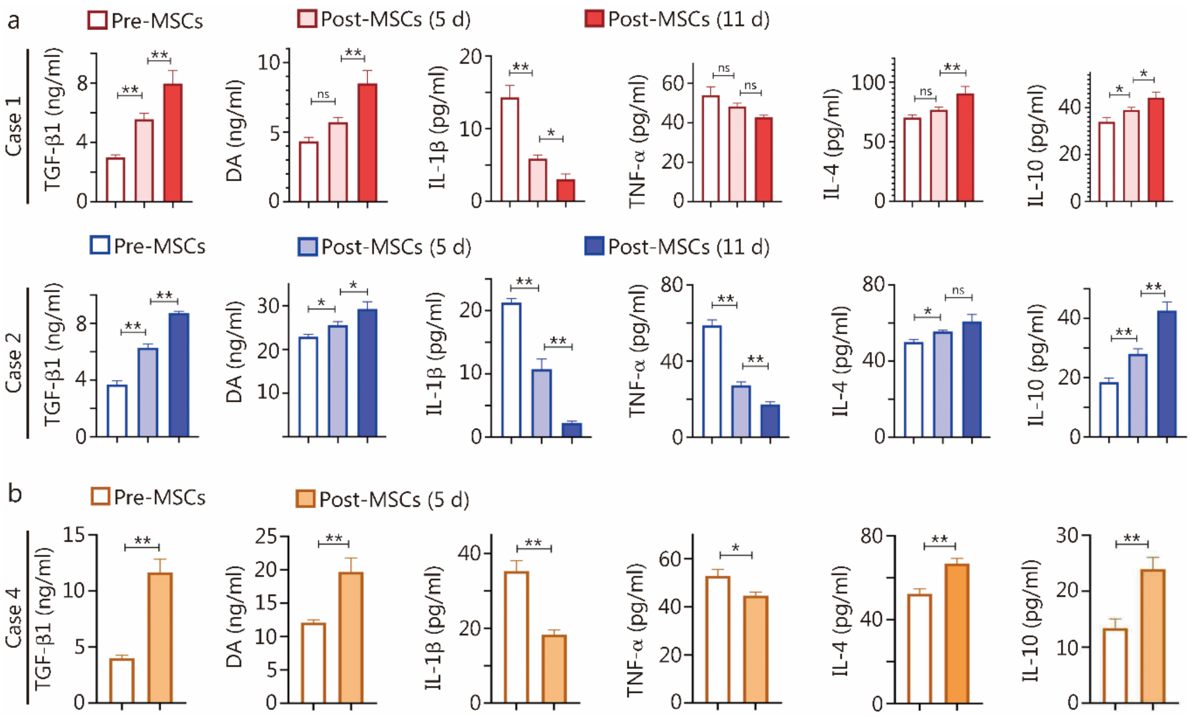

Fig. S7 Phase I hOM-MSC implantation clinical trial for PD patients, related to Fig. 7. **a** ELISA showing a histogram of TGF-β1, DA, IL-1β, TNF-α, IL-4, and IL-10 protein concentration in serum of case 1/2 before hOM-MSCs transplantation and 5 and 11 d after transplantation. **b** ELISA showing a histogram of TGF-β1, DA, IL-1β, TNF-α, IL-4, and IL-10 protein concentration in serum of case 4 before hOM-MSCs transplantation and 5 d after transplantation. Data are represented as mean ± SEM. $^{*}P < 0.05$, $^{**}P < 0.01$, ns non-significant, Pre-MSCs previous treatment olfactory mucosa mesenchymal stem cells, Post-MSCs post-treatment olfactory mucosa mesenchymal stem cells, hOM-MSCs hypoxia-olfactory mucosa mesenchymal stem cells, PD Parkinson's disease, DA dopamine, TNF-α tumor necrosis factor-α, IL-4 interleukin-4, IL-10 interleukin-10, IL-1β interleukin-1β
