# Supplementary material for: TGF-β1 mediates hypoxia-preconditioned olfactory mucosa mesenchymal stem cells improved neural functional recovery in Parkinson’s disease models and patients
Source: Mil Med Res. 2024 Jul 22;11:48. doi: 10.1186/s40779-024-00550-7 (PMC11265117; doi:10.1186/s40779-024-00550-7)
Supplement: Supplementary file 2 — Additional file 2: Table S1 The characteristics of the patients. Table S2 Neurological function score in case 1. Table S3 Neurological function score in case 2. Table S4 Neurological function score in case 3. Table S5 Neurological function score in case 4. Table S6 Neurological function score in case 5. [file 40779_2024_550_MOESM2_ESM.pdf]

Table S1 The characteristics of the patients

Characteristics	Value and method
Gender	2 females, 3 males
Age (years)	69.2 (62 – 79)
Number of cell transplants per course of treatment	2 – 3
The number of cells transplanted per transplant	5×10^7
Cell transplantation method	Intraspinal injection
Hospital length of stay (d)	19.6 (14 – 21)

Table S2 Neurological function score in case 1

Compound	Pre-treatment	1 month	6 months
Mental, behavioral and emotional	6	3	4
Activities of daily living	21	14	18
Motion examination	48	31	43
Complications of treatment	4	3	4
Total UPDRS score	79	51	69
Hoehn and Yahr rating scale	3	2	2.5
Schwab and England daily activity scale (%)	50	70	60

Table S3 Neurological function score in case 2

Compound	Pre-treatment	1 month	6 months
Mental, behavioral and emotional	5	3	5
Activities of daily living	24	16	19
Motion examination	56	40	52
Complications of treatment	2	2	2
Total UPDRS score	89	61	78
Hoehn and Yahr rating scale	3	2	3
Schwab and England daily activity scale (%)	50	70	60

Table S4 Neurological function score in case 3

Compound	Pre-treatment	1 month	6 months
Mental, behavioral and emotional	4	2	3
Activities of daily living	21	14	19
Motion examination	52	39	54
Complications of treatment	4	4	4
Total UPDRS score	85	59	80
Hoehn and Yahr rating scale	3	2	3
Schwab and England daily activity scale (%)	50	70	50

Table S5 Neurological function score in case 4

Compound	Pre-treatment	1 month	6 months
Mental, behavioral and emotional	6	1	3
Activities of daily living	32	13	20
Motion examination	58	39	54
Complications of treatment	5	2	4
Total UPDRS score	101	55	81
Hoehn and Yahr rating scale	4	2	3
Schwab and England daily activity scale (%)	30	60	50

Table S6 Neurological function score in case 5

Compound	Pre-treatment	1 month	6 months
Mental, behavioral and emotional	5	3	4
Activities of daily living	18	12	16
Motion examination	49	35	47
Complications of treatment	3	3	3
Total UPDRS score	75	53	70
Hoehn and Yahr rating scale	3	2	3
Schwab and England daily activity scale (%)	50	70	60
